# A probabilistic sampling strategy for estimating plant density in *Posidonia oceanica* meadows

**DOI:** 10.1007/s10661-025-13973-z

**Published:** 2025-04-11

**Authors:** Alice Bartolini, Agnese Marcelli, Rosa Maria Di Biase, Lorenzo Fattorini, Silvia Ferrini

**Affiliations:** 1 Department of Economics and Management, University of Trento, Via Inama 5, 38122 Trento, TN Italy; 2 Department of Political Economics and Statistics, University of Siena, Piazza S. Francesco, 53100 Siena, SI Italy; 3 Department of Political and International Sciences, University of Siena, Via P.A. Mattioli, 10, 53100 Siena, SI Italy; 4 Centre for Social and Economic Research on the Global Environment (CSERGE), School of Environmental Sciences, University of East Anglia, Norwich, UK; 5 University College London, London, UK

**Keywords:** *Posidonia oceanica*, Ecosystem accounting, Design-based inference, Simulation study

## Abstract

Marine and coastal ecosystems, such as seagrasses, mangroves, and coral reefs, provide a range of essential provisioning, regulating and cultural ecosystem services. Recent United Nations guidelines on ecosystem accounting (SEEA EA) emphasise the need for biophysical data as the foundation for compiling ecosystem accounts and conducting economic evaluations for developing indicators and informing policies and interventions. However, data availability on marine ecosystems is limited with respect to terrestrial ones. Moreover, the collection of biophysical data on marine ecosystem extent and condition required for ecosystem accounting (EA) is often not aligned with existing habitat monitoring strategies. This study aims to address the scarcity of spatial data on marine ecosystems and facilitate the integration of current monitoring strategies with the scope of EA. We propose the application of design-based inference for the estimation, mapping, and monitoring of key ecological attributes of marine ecosystems. We focus on the habitat of *Posidonia oceanica*, an endemic seagrass of the Mediterranean Sea, but the proposed strategy is adaptable to other ecosystems. The benefits of appropriate probabilistic sampling schemes for assessing *P. oceanica* are explored via simulation testing. The performance of different sample schemes in artificial populations reveals that reliable estimates of density (as well as their precision) can be obtained even with low sample sizes. The empirical viability of our methodology is exemplified using data collected on a meadow located in an Italian Marine Protected Area (Puglia region, Southern Italy).

**Supplementary Information:**

The online version contains supplementary material available at 10.1007/s10661-025-13973-z.

## Introduction

*Posidonia oceanica* (L.) Delile is an endemic seagrass of the Mediterranean Sea that provides numerous ecosystem services (ES), including carbon sequestration, coastal protection, marine-based tourism, and recreation (Bartolini et al., 2024; Campagne et al., 2015; Leduc et al., 2023; Monnier et al., 2021, 2022; Pergent-Martini et al., 2021). The condition of *P. oceanica* is often used as an indicator of the general ecological status of the marine ecosystem (Bellissimo et al., 2020; Romero et al., 2007). At the same time, anthropogenic activities contribute to its decline (Bakirman & Gumusay, 2020; Hinz et al., 2024; Pergent-Martini et al., 2022), making its conservation a priority under the EU Habitats Directive (92/43/EEC, 1992) and the Marine Strategy Framework Directive (MSFD, 2008/56/EC).

The policy demand for biophysical data on the state of *P. oceanica* and other marine ecosystems extends beyond international directives and includes recent advances and requirements in ecosystem accounting (EA). The move towards mandatory reporting on ecosystem accounts (European Commission, 2024; Joint Research Centre, 2024) underlines the importance of collecting reliable biophysical information, representing the backbone of informative accounts and their economic value, thereby supporting effective coastal management and habitat conservation (Borja et al., 2024; Chen & Vardon, 2024; Nazombe et al., 2024).

In this context, the paper proposes a methodology for collecting and analysing biophysical data related to the habitat of *P. oceanica*. This methodology aims to (i) be statistically robust and thus ensure reliable estimates as well as associated measures of uncertainty, (ii) be readily scalable and adaptable to different ecosystems and habitats, and (iii) align with national and international reporting and monitoring directives requirements. As a key reporting framework, the United Nations et al (2021) recently adopted the System of Environmental Economic Accounting – Ecosystem Accounting (SEEA EA). The SEEA EA organises biophysical data on ecosystems, measuring and valuing ecosystem assets and services, and integrating this information with the economic system. The SEEA EA establishes five core accounts that facilitate the inclusion of ecosystems into the System of National Accounts (SNA). These accounts are categorised into stock accounts, which record the physical extent and condition of ecosystem assets and track their changes over time, and flow accounts, which quantify the provision of ES in both physical and monetary terms. Finally, the monetary asset accounts show the economic value of each ecosystem asset.

Accurate biophysical information is essential for the correct compilation of accounts. The extent (total area) and condition (quality) of an ecosystem determine its capacity to provide ecosystem services. Despite this, examples of economic valuation of ES provided by *P. oceanica,* which take into account meadow conditions, are scarce (Addamo & La Notte, 2023; Capasso et al., 2023; Pisani et al., 2024).

This paper contributes to filling this gap by proposing a probabilistic sampling design to estimate, map, and monitor over time *P. oceanica* ecological attributes, thereby ensuring the reliable collection of data critical for effective reporting on ecosystem accounts. The main advantage of design-based inference is its objectivity, as the precision of the estimators stems from the sampling scheme actually adopted in the survey, without relying on model assumptions (Särndal et al., 1992). The risks associated with using non-probabilistic samples in ecology are a long-debated issue. Boyd et al. (2023) recently highlighted the potential for incorrect generalisations and biased estimates, which should be acknowledged and mitigated whenever possible. Balestri et al. (2003), referring to the spatial variation of the habitat *P. oceanica*, emphasised (i) the importance of sampling at multiple scales when studying this habitat and (ii) the need to carefully select sampling procedures to draw accurate inferences about its status and trends. The need for the use of statistical sampling for reliable and accurate estimates of the health status of ecosystems has been highlighted in several EA applications, including water quality (Bagstad et al., 2020).

This work also contributes to aligning EA requirements with current monitoring strategies, which are not designed for reporting purposes, resulting in inadequate data for proper national-level reporting on EA. Such inconsistency primarily stems from the scale at which information is collected, which represents an important aspect of EA (Costa et al., 2024). Monitoring data are often collected on sampling stations distributed across *P. oceanica* meadows, with assessments conducted at regional or sub-regional levels. However, the knowledge about characterising areas outside these stations is limited, and data are still too scarce even to meet international directives’ requirements (Valette-Sansevin et al., 2019). For EA purposes, the spatial distribution of indicators reflecting the condition of ecosystems needs to be available at larger scales (e.g., national level). Although sampling stations are selected to be representative of the meadow (see, for example, the ISPRA monitoring protocol, available at www.isprambiente.gov.it), to the best of our knowledge, no studies have adopted probabilistic sampling schemes to make inferences outside these stations.

We focus on the shoot density of *P. oceanica* meadows since it is a commonly measured parameter in monitoring activities, representing a valid indicator of the habitat health status and is used to build several ecological indexes (Montefalcone, 2009; Tursi et al., 2022). However, the proposed strategy is highly adapted to different scales, attributes, and ecosystems, particularly marine ones, thus responding to the urgent need to face the scarcity of special data on them (Addamo et al., 2024).

The “Materials and methods” section of the paper introduces the proposed methodology. The theoretical basis for the density estimation and mapping is provided in the “Theory for probabilistic density estimation, mapping and monitoring” section. We test our proposal through a simulation study on artificial populations with different spatial patterns (the “Simulation study” section and the “Results from the simulation study” section), and we deploy real data collected by traditional method on a meadow located in the Tremiti Islands Marine Protected Area (Puglia Region, South Italy) and assess the potential of using our probabilistic sampling scheme instead (the “Case study” section). The “Conclusions” section concludes.

## Materials and methods

In this section, we propose two probabilistic sampling schemes—uniform random sampling and tessellation-stratified sampling—along with two interpolation methods—nearest neighbour and inverse distance weighting—for estimating, mapping, and monitoring *P. oceanica* density. First, we present the theoretical foundation of these methods. Then, we perform a simulation study to validate our approach and assess how the different sampling schemes and interpolation methods perform under varying *P. oceanica* density distributions. Results from such simulation study provide the theoretical basis for the empirical application presented in the “Case study” section.

### Theory for probabilistic density estimation, mapping, and monitoring

Let $A$ be a delineated *P. oceanica* meadow of size $\left|A\right|$, where a finite (even if possibly large) population $U$ of $N$ plants is settled, identified by the first $N$ integers, i.e., $U=\left\{1,\dots ,N\right\}$. Any plant $j\in U$ is characterised by its location ${c}_{j}\in A$.

Let $Y$ be a dummy variable that for each plant $j\in U$ takes the value ${y}_{j}=1$ in such a way that the population size $N$ can be expressed as the total of $Y$ over $U$, i.e.,

1 $$N=\sum \nolimits_{j\in U}{y}_{j}$$

Our target is to provide an objective evaluation of the plant density

2 $$D=N/\left|A\right|$$

As the meadow size is usually known, the statistical problem is the estimation of the population size $N$. For this purpose, it is worth noting that the discrete summand (1) can be approximately rewritten as the integral over $A$ of the function

3 $$g\left(p;a\right)=\frac{1}{a}\sum \nolimits_{j\in U}{y}_{j}{z}_{j}\left(p\right) ,$$

where for each plant $j\in U$, ${z}_{j}\left(p\right)$ is the dichotomous function equal to 1 if the plant lies within the oriented quadrat of size $a$ centred at $p$, and is equal to 0 otherwise. In practice, $g\left(p;a\right)$ gives the density of plants within the quadrat plot of size $a$ centred at $p$, in such a way that whatever $a$,

4 $$T\cong {\int }_{A}g\left(p;a\right)dp .$$

Without entering the technical details, which have been well explained in Gregoire and Valentine (2008, section 7.5), it should be noticed that the integral (4) is slightly smaller than $T$ owing to the presence of plants near the meadow edges. However, the error is negligible if the quadrat size $a$ is much smaller than the meadow size $\left|A\right|$. Owing to (4), the estimation of $N$ can be suitably treated as a Monte Carlo integration (see e.g., Gregoire & Valentine, 2008; Mandallaz, 2008).

For monitoring purposes, consider 2 years ${t}_{1}<{t}_{2}$ and let ${U}_{1}$ and ${U}_{2}$ be the populations of plants at times ${t}_{1}$ and ${t}_{2}$ and ${y}_{1,j}$ and ${y}_{2,j}$ are the values of the dummy variable $Y$ for the plants of the two populations. We are interested in the change of the population sizes (or densities, indifferently).

5 $${\Delta }_{N}={N}_{2}-{N}_{1}=\sum \nolimits_{j\in {U}_{2}}{y}_{2,j}-\sum \nolimits_{j\in {U}_{1}}{y}_{1,j}$$

Owing to (4), the difference (5) can be approximated by

6 $${\Delta }_{N}\cong {\int }_{A}{g}_{2}\left(p;a\right)dp-{\int }_{A}{g}_{1}\left(p;a\right)dp={\int }_{A}\delta \left(p;a\right)dp ,$$

where ${\delta \left(p;a\right)=g}_{2}\left(p;a\right)-{g}_{1}\left(p;a\right)$ and ${g}_{1}\left(p;a\right)$ and ${g}_{2}\left(p;a\right)$ are the functions of type (3) at the 2 years ${t}_{1}$ and ${t}_{2}$, so that even the estimation of change ${\Delta }_{N}$ can be treated as Monte Carlo integration.

To estimate population sizes or their changes, we can adopt the basic uniform random sampling (URS) that consists in randomly and independently selecting $R$ sample locations onto $A$. As the random selection may provide unequal coverage of meadows, we propose the alternative use of tessellation stratified sampling (TSS), applied for the first time in the 2005 Italian National Forest Inventory (Fattorini et al., 2006), followed some years later by U.S. Department of Agriculture (Tomppo et al., 2010). The TSS scheme consists of partitioning $A$ into $R$ tassels of equal size and randomly selecting one sample location in each tassel.

Subsequently, at any selected location ${p}_{i} (i=1,\dots ,R)$ a quadrat^1^ of size ${a}_{0}$ is centred at the location and oriented in the same direction for all locations. The plant density $g\left({p}_{i};{a}_{0}\right)$, henceforth denoted for brevity as ${g}_{i}$, is recorded. Then, the Monte Carlo estimator of $D$ is given by

7 $${\widehat{\overline{D}}}_{(R)}=\frac{1}{R}\sum \nolimits_{i=1}^{R}{g}_{i} .$$

If the size ${a}_{0}$ of the quadrat adopted to record densities at selected locations is much smaller than $A$, the Monte Carlo estimator (7) is approximately unbiased, normal, and consistent under URS and TSS. Moreover, under TSS, the estimator (7) is super-efficient with respect to URS, as its variance decreases with $R$ at a rate faster than ${R}^{-1}$ (see, e.g., Barabesi & Franceschi, 2011; Barabesi et al., 2012).

Regarding the estimation of the precision, it is customary to estimate the sampling variance of (7) from the variance of the densities recorded at the $R$ points, i.e.,

8 $${v}_{R}^{2}=\frac{1}{R\left(R-1\right)}\sum \nolimits_{i=1}^{R}{\left({g}_{i}-{\widehat{\overline{D}}}_{R}\right)}^{2},$$

which turns out to be unbiased under URS and conservative under TSS. Therefore, owing to the normality of (7) under the two schemes

9 $${\widehat{\overline{D}}}_{R}\pm 2{v}_{R}$$

is a confidence interval with coverage equal to or greater than 95% under URS or TSS, respectively.

In the last decade, there was an increasing interest in making inference not only for totals of ecological attributes (as the density is), but also in their spatial distributions throughout the study region. Traditionally, maps are predicted by means of model-based methodologies, such as geostatistical techniques, based on several (sometimes unrealistic) assumptions on the phenomena to be mapped (e.g., Cressie, 1993). Recently, mapping has been approached from a model-assisted point of view, performing estimation on the basis of the very simple Tobler law, for which the values of the interest variables in neighbouring locations are more similar than those at locations that are far apart (Tobler, 1970). See Fattorini et al., (2018a, b) for details on model-assisted mapping and related motivations.

Regarding the mapping of the plant density $g\left(p;{a}_{0}\right)$ within the oriented quadrat of size ${a}_{0}$ centred at any $p\in A$, quoting from Fattorini et al. (2018b), we adopt the inverse distance weighting (IDW) interpolator

10 $$\widehat{g}\left(p;{a}_{0}\right)=\frac{\sum_{i=1}^{r}{g}_{i}{\Vert {p}_{i}-p\Vert }^{-\alpha }}{\sum_{i=1}^{r}{\Vert {p}_{i}-p\Vert }^{-\alpha }} ,$$

where $\Vert {p}_{i}-p\Vert$ denotes the Euclidean distance and $\alpha >2$ is a smoothing parameter that can be arbitrarily prefixed or chosen in a complete data-driven framework by leave-one-out techniques (Fattorini et al., 2023). To avoid the $\alpha$ choice, we alternatively use the nearest-neighbour (NN) interpolator (Fattorini et al., 2022)

11 $$\widehat{g}\left(p;{a}_{0}\right)=g\left({p}_{nn\left(p\right)};{a}_{0}\right) ,$$

where $nn\left(p\right)={argmin}_{i=1,\dots ,R}\Vert {p}_{i}-p\Vert$ is the sample location that is nearest to $p$.

While the Tobler model is exploited to justify the interpolators (10) and (11) that both give importance to the sample observations near to the point $p$ at which interpolation occurs, the properties of (10) and (11) stem only from the sampling scheme adopted to select the sample locations in a complete design-based perspective, without any assumption on the population to be interpolated. Fattorini et al., (2022, 2023) prove that under URS and TSS, the two interpolators are asymptotically design-unbiased and consistent, irrespective of the fact that the Tobler law may be valid or not.

Regarding the estimation of precision, in accordance with Fattorini et al., (2022, 2023), we use the interpolated maps $\left\{\widehat{g}\left(p;{a}_{0}\right), p\in A\right\}$ as pseudo populations from which bootstrap samples are selected by means of the same spatial scheme (URS or TSS) adopted to select the original sample of locations. Because, owing to consistency, the estimated maps converge to true ones, the bootstrap distributions of the interpolators (10) and (11) achieved from resampling from these maps should converge to the true distributions, also providing reliable estimators of their mean squared errors. Accordingly, for each $p\in A$ the pseudo population bootstrap estimator of the root mean squared error of $\widehat{g}\left(p;{a}_{0}\right)$ is given by

12 $${\widehat{rmse}}_{M}^{*}\left(p;{a}_{0}\right)={\left[\frac{1}{M}\sum \nolimits_{m=1}^{M}{\left\{{\widehat{g}}_{m}^{*}\left(p;{a}_{0}\right)-\widehat{g}\left(p;{a}_{0}\right)\right\}}^{2}\right]}^\frac{1}{2},$$

where ${\widehat{g}}_{m}^{*}\left(p;{a}_{0}\right)$ is the bootstrapped value of the IDW or NN interpolator at $p\in A$ achieved from the $m$ th bootstrap sample. Obviously, the map of $\widehat{g}\left(p;{a}_{0}\right)$ and the corresponding map of precisions ${\widehat{rmse}}_{M}^{*}\left(p;{a}_{0}\right)$ cannot be given for any point $p$ of the uncountable continuous set $A$, but for a network of points sufficiently dense to ensure a good definition of the resulting pictures.

As for monitoring purposes, we are interested in the estimation of the density change (or size change indifferently) at the two times ${t}_{1}<{t}_{2}$. As previously stated in (6), the difference ${\Delta }_{N}$ can be expressed as the integral onto $A$ of the difference ${\delta \left(p;{a}_{0}\right)=g}_{2}\left(p;{a}_{0}\right)-{g}_{1}\left(p;{a}_{0}\right)$. Therefore, the Monte Carlo estimator of density change ${\Delta }_{D}$ under URS or TSS is given by

13 $${\widehat{\Delta }}_{D,R}=\frac{1}{R}\sum \nolimits_{i=1}^{R}{\delta }_{i},$$

where ${{\delta }_{i}=g}_{2}\left({p}_{i};{a}_{0}\right)-{g}_{1}\left({p}_{i};{a}_{0}\right)$ for $i=1,\dots ,R$ are the density changes recorded at the R sample locations visited at times ${t}_{1}$ and ${t}_{2}$. The variance estimator is given by

14 $${v}_{R}^{2}=\frac{1}{R(R-1)}\sum \nolimits_{i=1}^{R}{({\delta }_{i}-{\widehat{\Delta }}_{D,R})}^{2},$$

which once again is unbiased under URS and conservative under TSS, so that the confidence interval

15 $${\widehat{\Delta }}_{D,R}\pm 2{v}_{R}$$

has coverage equal to or greater than 95% under URS or TSS, respectively. Similarly, the mapping of the density change throughout the meadow and the bootstrap estimation of precision is performed by means of Eqs. (9), (10), and (11) with the density $g(p;{a}_{0})$ replaced by the density change $\delta (p;{a}_{0})$.

### Simulation study

To empirically evaluate our proposal, a simulation study was designed on four artificial populations settled on a rectangular region of size $700 \times 500$ m^2^ and sampled by $R=25, 36, 49, 64$ oriented quadrat plots of 0.16 m^2^ randomly located in the region in accordance with the URS and TSS sampling schemes. For mapping purposes, we considered both NN and IDW interpolation methods. The area size of 35 hectares represents the average size of an Italian *P. oceanica* meadow, retrieved from the Mediterranean seagrass map (Telesca et al., 2015, accessed in April 2024). The plot size and the number of plots align with several *P. oceanica* monitoring protocols, including the one provided by the Italian Institute for Environmental Protection and Research (ISPRA, available at www.isprambiente.gov.it). The analysis has been conducted in R (R Core Team, 2023).

The four populations represent different spatial patterns observable in seagrass meadows of *P. oceanica*, i.e., the spatial distribution of shoots—identified by points—over the study region. To this purpose, we distributed $N=87.5$ million points according to four different spatial patterns identified from the literature (Paticchio, 2013; Rouanet et al., 2023), for a density of $D=250$ shoots/m^2^. The four patterns were referred to as regular, trended, clustered, and striped. The density was derived from the average density of shoots/m^2^ obtained from ISPRA monitoring data collected within the Marine Strategy Framework Directive (accessed in April 2024).

**Fig. 1:**
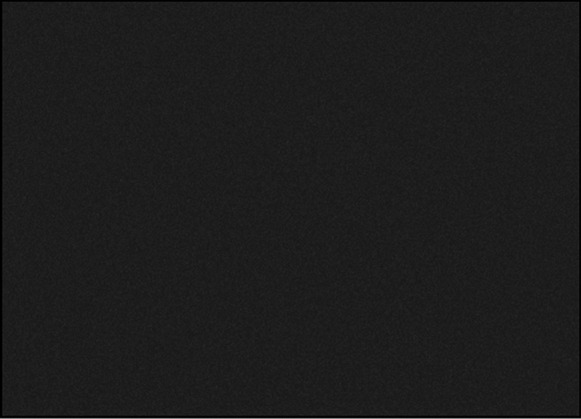
Population 1. The 87.5 million points uniformly distributed over the rectangular area of size 35 hectares

**Fig. 2:**
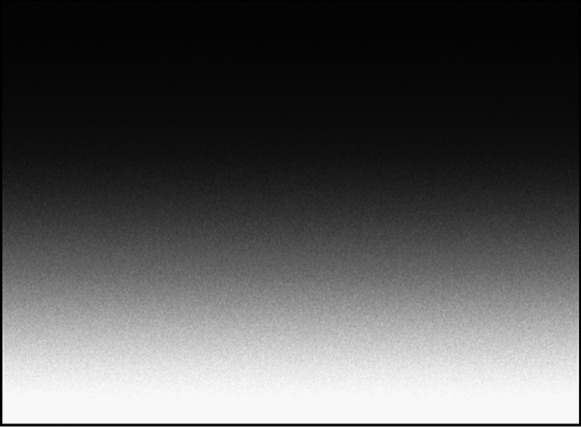
Population 2. The 87.5 million points distributed according to a trended spatial pattern over the rectangular area of size 35 hectares

**Fig. 3:**
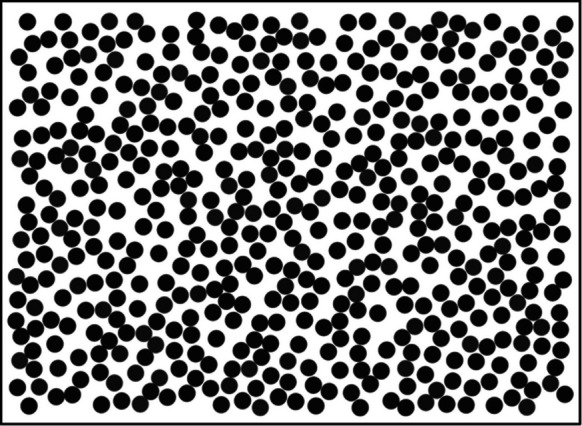
Population 3. The 87.5 million points distributed according to a clustered spatial pattern over the rectangular area of size 35 hectares

**Fig. 4:**
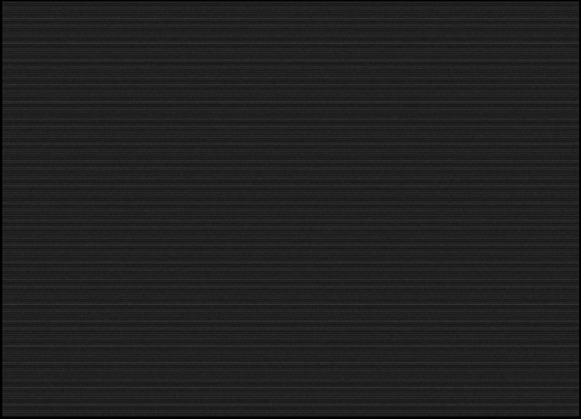
Population 4. The 87.5 million points distributed according to a striped spatial pattern over the rectangular area of size 35 hectares

For the regular pattern, we generated the 87.5 million points of coordinates $x$ and $y$, where $x$ and $y$ were random numbers uniformly distributed on $(0, 700)$ and $(0, 500)$, respectively. For the trended pattern, we generated the 87.5 million^2^$x$ coordinates in the same way as for the regular pattern, i.e., as random numbers uniformly distributed on $(0, 700)$ and $y$ coordinates as:

$$y=\left(1-{u}^{1/3}\right)\cdot 500,$$

where $u$ was randomly generated from a uniform distribution on $(\text{0,1})$. For the clustered pattern, we generated 500 points, i.e., the centres of the clusters, uniformly distributed on the rectangle, but discarding and re-generating those having a minimum distance smaller than 20 m from the rectangle edges and from each other. The 87.5 million points were then allocated around the clusters’ centres by randomly generating a radius from a uniform distribution on (0, 10) and an angle from a uniform distribution on (0, 2π). We define the cluster size as the number of points in each cluster. The sizes were chosen to ensure a shoot density/m^2^ within the clusters that did not exceed the maximum density/m^2^ reported in the ISPRA data. Specifically, we allocated the cluster sizes as follows: 50 clusters were assigned a size of 260,000 points each, 50 clusters with 250,000 points each, 50 with 230,000 points each, 50 with 215,000 points, 50 with 190,000 points, 50 with 170,000 points, 50 with 150,000 points, 50 with 115,000 points, 51 with 90,000 points, and 50 clusters with 80,000 points. This distribution ensured a diverse range of cluster sizes while adhering to the specified density constraints. Finally, for the striped pattern, we considered an average width of 1.5 m for *P. oceanica* stripes, interspersed by stripes of 0.75 m width, as reported in Rouanet et al. (2023). The $x$ coordinates were generated as for the regular pattern, while $y$ coordinates were generated as random numbers uniformly distributed within the 1.5 m wide intervals. Points with $y$ coordinates outside these intervals were discarded and re-generated to ensure they fall within the defined *P. oceanica* stripes. The resulting populations are reported in Figs. 1, 2, 3, and 4.

**Figure 5:**
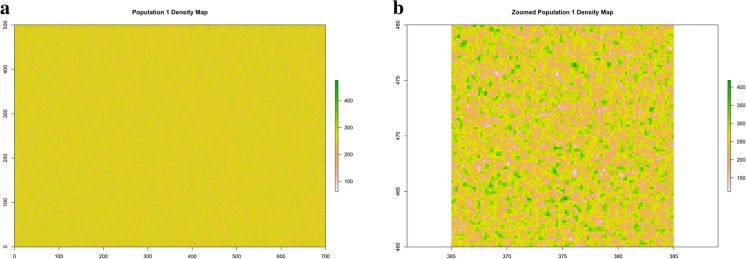
**a** Population 1. Density map of the regular population. **b** Population 1. Zoomed density map in a 20 × 20 m quadrat within the rectangle

**Figure 6:**
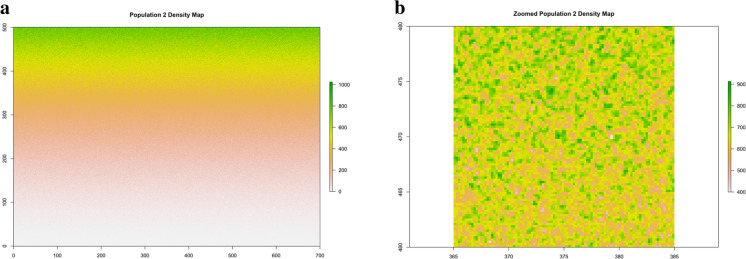
**a** Population 2. Density map of the trended population. **b** Population 2. Zoomed density map in a 20 × 20 m quadrat within the rectangle

**Figure 7:**
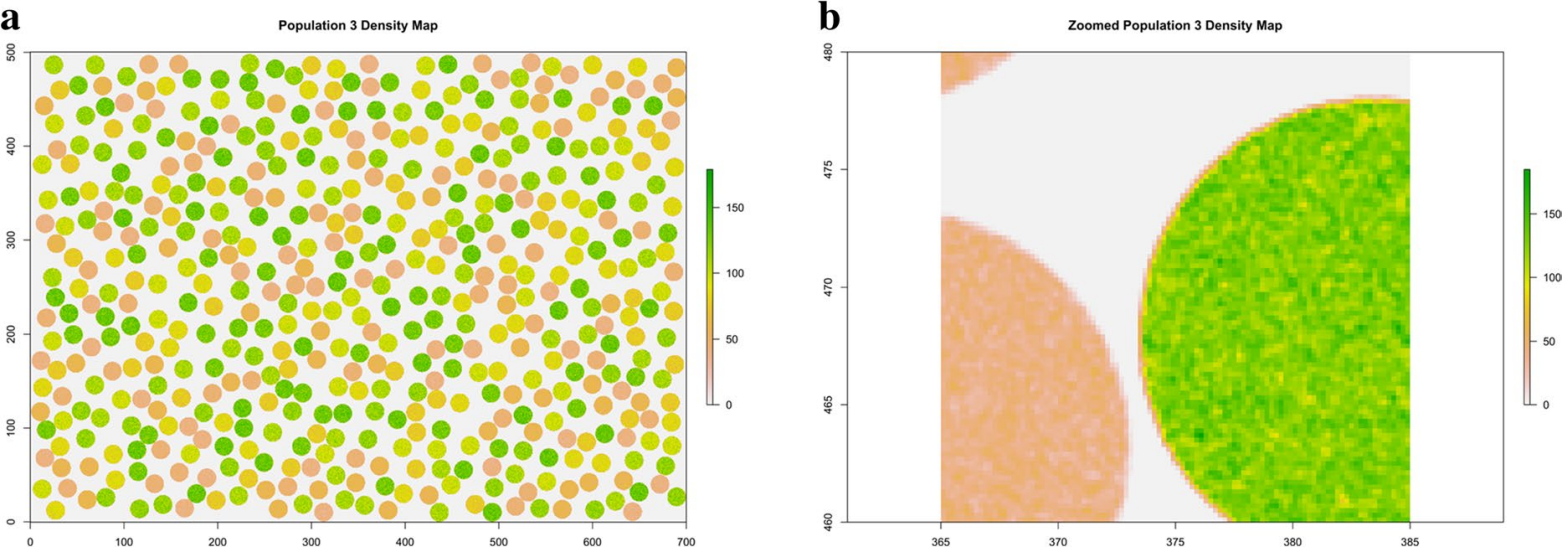
**a** Population 3. Density map of the clustered population. **b** Population 3. Zoomed density map in a 20 × 20 m quadrat within the rectangle

**Figure 8:**
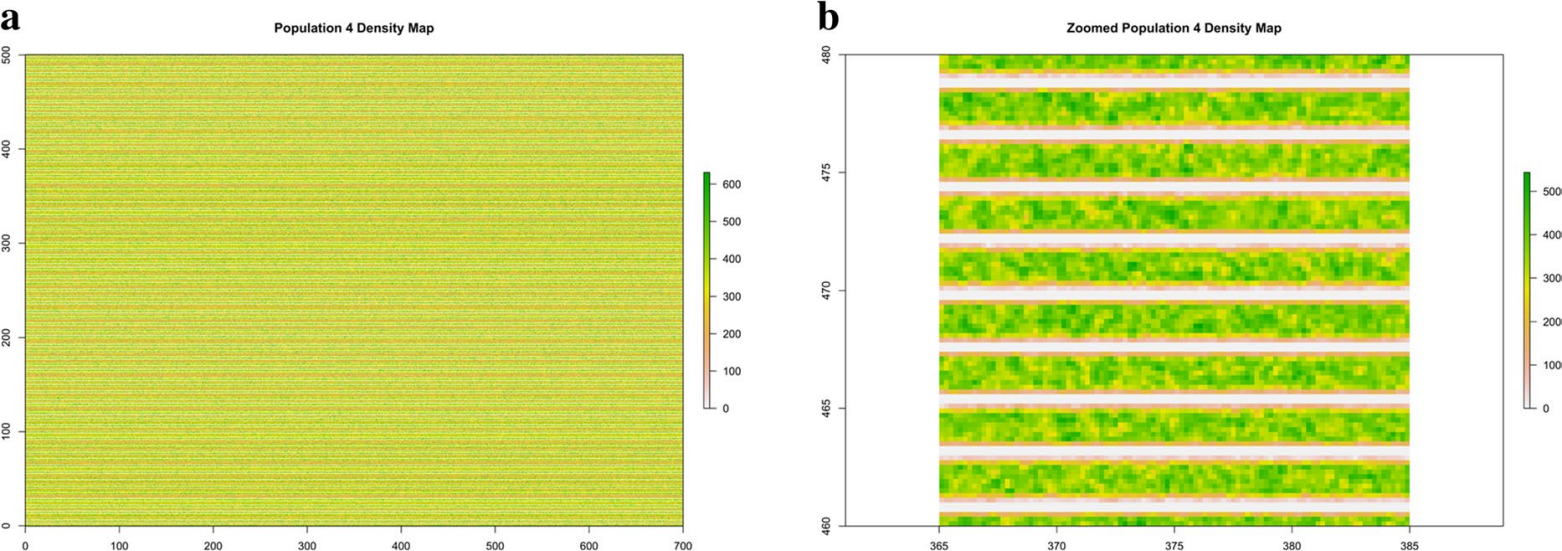
**a** Population 1. Density map of the stripped population. **b** Population 4. Zoomed density map in a 20 × 20 m quadrat within the rectangle

Because there is a density defined at each point of the rectangular region within the oriented quadrat of size 0.16 m^2^ centred at the point (see Eq. 3), we also considered the density maps of the four populations. To have an accurate representation of density, we computed densities at each node of a 0.2 m grid. The resulting maps are reported in Figs. 5, 6, 7, and 8.

For each population and each sampling scheme (URS and TSS), $R=25, 36, 49, 64$ locations were selected, and, at each location, the number of shoots lying within the oriented quadrat of size 0.16 m^2^ centred at the location was recorded to determine the density. Given the time needed to compute the Monte Carlo estimator (7) from 87.5 million points, we instead used density values from the maps in Figs. 5, 6, 7, and 8. For each location, we assigned the density of the nearest node on the 0.2 m grid. We tested both methods and confirmed that the results were identical.

For each combination of population, sampling scheme and sample size, we independently replicated the procedure $M = \text{10,000}$ times, at each computing the Monte Carlo estimator (7), the sampling variance (8), and the 95% confidence interval (9). Then, from the set of the $M$ density estimates, we empirically computed: (i) the expectation

16 $$E=\frac{1}{M}\sum \nolimits_{m=1}^{M}{\widehat{\overline{D}}}_{\left(R\right),m}$$

where ${\widehat{\overline{D}}}_{\left(R\right),m}$ is the density estimate computed by (7) at the $m$ th simulation run, from which we derived the bias

17 $$B=E\left({\widehat{\overline{D}}}_{(R)}\right)-D$$

and the relative bias

18 $$RB =\frac{B}{D}$$

(ii) the mean squared error

19 $$\text{MSE}=\frac{1}{M}\sum \nolimits_{m=1}^{M}{\left({\widehat{\overline{D}}}_{\left(R\right),m}-D\right)}^{2}$$

from which we derived the root mean squared error

20 $$\text{RMSE}=\sqrt{\text{MSE}}$$

and the relative root mean squared error

21 $$\text{RRMSE}=\frac{\text{RMSE}}{D}$$

(iii) the coverage of the 95% confidence intervals

22 $$C=\frac{1}{M}\sum \nolimits_{m=1}^{M}I\left({\widehat{\overline{D}}}_{\left(R\right),m}-2{v}_{R,m}<D<{\widehat{\overline{D}}}_{\left(R\right),m}+2{v}_{R,m}\right)$$

where ${v}_{R,m}^{2}$ is the variance estimate computed by (8) at the $m$ th simulation run and $I$ is the indicator function.

For each combination of population, sampling scheme and sample size, at each replication, we estimated the density map adopting both the NN interpolator (Eq. 9) and the IDW interpolator (Eq. 10) with the smoothing parameter pre-fixed at $\alpha =4$ to avoid very time-consuming data-driven choices. At each replication, we performed interpolation onto the $K=\text{14,000}$ nodes of a 5 m grid and we compared the resulting $M$ maps to the artificial ones. To this purpose, for each $k$ th node ${p}_{k}$ of the 5 m grid, we computed: (i) the expectation

23 $$e\left({p}_{k}\right)=\frac{1}{M}\sum \nolimits_{m=1}^{M}{\widehat{g}}_{m}\left({p}_{k}; 0.16\right), k=1,\dots ,K$$

where ${\widehat{g}}_{m}\left({p}_{k}; 0.16\right)$ is the interpolated density computed by (9) or (10) at the $m$ th simulation run, from which we derived the bias

24 $$b\left({p}_{k}\right)=e\left({p}_{k};0.16\right)-g\left({p}_{k};0.16\right), k=1,\dots ,K$$

(ii) the root mean squared error

25 $$rmse\left({p}_{k}\right)=\sqrt{\frac{1}{M}\sum \nolimits_{m=1}^{M}{\left[{\widehat{g}}_{m}\left({p}_{k}; 0.16\right)-g\left({p}_{k};0.16\right)\right]}^{2}} , k=1,\dots ,K.$$

Finally, for each combination of population, sampling scheme, sample size, and interpolator, we synthesised bias values and root mean squared errors by their averages over the grid nodes, i.e.,

26 $$\overline{bias }=\frac{1}{K}\sum \nolimits_{k=1}^{K}b\left({p}_{k}\right)$$

27 $$\overline{rmse }=\frac{1}{K}\sum \nolimits_{k=1}^{K}rmse\left({p}_{k}\right)$$

### Results from the simulation study

Tables 1 and 2 report the simulation results for the four populations. Table 1 summarises the results regarding the density estimation, showing the relative bias ($RB$), the root relative mean squared error ($\text{RRMSE}$), and the coverage of the 95% confidence intervals ($C$) for each combination of population, sample size, and sampling scheme. As the sample size increases, both $RBs$ and $\text{RRMSEs}$ quickly decrease for both the sampling schemes, while $Cs$ improve. For population 1, URS and TSS perform equivalently, owing to the regular spatial pattern of the shoots even with small sample sizes, while, in contrast, for population 2, which is characterised by a trended spatial pattern, the use of TSS significantly enhances the precision of the Monte Carlo density estimator. For population 3, we observe unsatisfying high $\text{RRMSE}$ values across all sample sizes and sampling schemes, indicating that the sampling effort is likely too small. In this case, the use of TSS does not provide substantial improvements. This also holds for population 4, where, similar to population 1, URS and TSS perform equivalently. However, $\text{RRMSE}$ values for population 4 are higher than those for population 1 but significantly lower than those for population 3, all remaining below 15%.

**Table 1:** Percent values of relative bias (RB), relative root mean squared error (RRMSE), and coverage of the 0.95 confidence interval (C95) reported for each combination of population, sample size, and sampling scheme

Sampling	*R*	Population 1			Population 2			Population 3			Population 4		
		${{R}}{{B}}{\%}$	${R}{R}{M}{S}{E}{\%}$	${{C}}95{\%}$	${{R}}{{B}}{\%}$	${R}{R}{M}{S}{E}{\%}$	${{C}}95{\%}$	${{R}}{{B}}{\%}$	${R}{R}{M}{S}{E}{\%}$	${{C}}95{\%}$	${{R}}{{B}}{\%}$	${R}{R}{M}{S}{E}{\%}$	${{C}}95{\%}$
URS	25	0.02	3.17	94.63	0.26	18.07	93.87	0.37	24.43	93.82	− 0.16	12.46	93.70
URS	36	− 0.03	2.69	94.49	0.05	15.04	94.46	0.41	20.59	93.99	− 0.12	10.34	94.70
URS	49	− 0.00	2.25	94.93	− 0.04	13.05	94.37	− 0.07	17.55	94.26	0.01	8.91	95.06
URS	64	− 0.02	1.97	95.25	− 0.23	11.44	94.55	0.18	15.37	94.77	− 0.06	7.78	95.31
TSS	25	− 0.05	3.15	94.61	0.01	5.05	100	− 0.10	24.53	93.56	− 0.25	12.61	93.87
TSS	36	− 0.04	2.66	94.38	− 0.08	3.79	100	0.01	20.26	94.21	− 0.07	10.45	94.28
TSS	49	− 0.04	2.26	94.59	0.02	3.01	100	0.03	17.43	94.84	0.01	9.01	94.53
TSS	64	− 0.01	2.00	94.97	− 0.06	2.52	100	− 0.00	15.28	95.03	0.04	7.84	94.86

**Table 2:** Average bias ($\overline{bias })$ and root mean squared error $(\overline{rmse })$ reported for any combination of population, sample size, sampling scheme, and interpolation method (NN, IDW)

Sampling	*R*	Population 1				Population 2				Population 3				Population 4			
		NN		IDW		NN		IDW		NN		IDW		NN		IDW	
		$\overline{{{B}} }$	$\overline{{R}{M}{S}{E} }$	$\overline{{{B}} }$	$\overline{{R}{M}{S}{E} }$	$\overline{{{B}} }$	$\overline{{R}{M}{S}{E} }$	$\overline{{{B}} }$	$\overline{{R}{M}{S}{E} }$	$\overline{{{B}} }$	$\overline{{R}{M}{S}{E} }$	$\overline{{{B}} }$	$\overline{{R}{M}{S}{E} }$	$\overline{{{B}} }$	$\overline{{R}{M}{S}{E} }$	$\overline{{{B}} }$	$\overline{{R}{M}{S}{E} }$
URS	25	0.08	39.74	0.08	29.64	− 4.81	84.95	− 5.06	71.54	4.73	395.43	4.68	334.56	0.41	157.21	0.36	117.57
URS	36	− 0.02	39.70	− 0.04	29.42	− 3.78	72.78	− 4.23	59.43	5.29	394.96	4.92	332.69	0.05	157.46	0.18	116.82
URS	49	0.05	39.79	0.04	29.23	− 2.91	64.35	− 3.48	51.00	3.77	393.58	3.52	330.54	− 0.09	157.52	− 0.11	116.15
URS	64	0.00	39.70	− 0.00	29.10	− 2.38	58.42	− 2.94	45.38	4.31	392.99	4.03	329.03	− 0.14	157.54	− 0.13	115.63
TSS	25	− 0.09	39.72	− 0.09	31.52	− 2.23	68.53	− 2.94	51.26	2.63	394.45	2.63	344.86	− 0.54	157.77	− 0.57	125.49
TSS	36	− 0.05	39.70	− 0.06	31.28	− 1.66	60.01	− 2.29	44.20	2.71	393.71	2.83	342.51	− 0.24	157.54	− 0.24	124.16
TSS	49	− 0.07	39.73	− 0.07	31.12	− 1.29	54.33	− 1.72	39.69	2.83	393.03	2.94	340.82	0.02	157.61	0.00	123.59
TSS	64	− 0.01	39.70	− 0.00	30.97	− 0.94	50.26	− 1.41	36.57	2.45	391.97	2.65	338.78	0.03	157.62	0.06	123.09

Table 2 reports the average bias ($\overline{bias })$ and average root mean squared error $(\overline{rmse })$, computed according to Eqs. (26) and (27), for each combination of population, sample size, sampling scheme, and interpolation method. For these combinations, we also report the maps of the bias values and of the root mean squared errors in the supplementary materials. The IDW interpolator generally performs better than the NN interpolator across all populations. Additionally, increasing the sample size enhances the accuracy of the interpolated maps, as theoretically proven by the consistency of the two interpolators under URS and TSS. Finally, for population 2, sample selection using TSS significantly improves the precision of both NN and IDW maps. For populations 1 and 4, we obtain similar $\overline{bias }$ and $\overline{rmse }$ values for the two sampling schemes. For population 3, TSS seems to improve $\overline{bias }$ values but not $\overline{rmse }$ ones. For this population, similar to what was reported for density estimation results, unsatisfying high $\overline{rmse }$ values are obtained, confirming too low sample effort also for mapping purposes.

## Case study

**Fig. 9:**
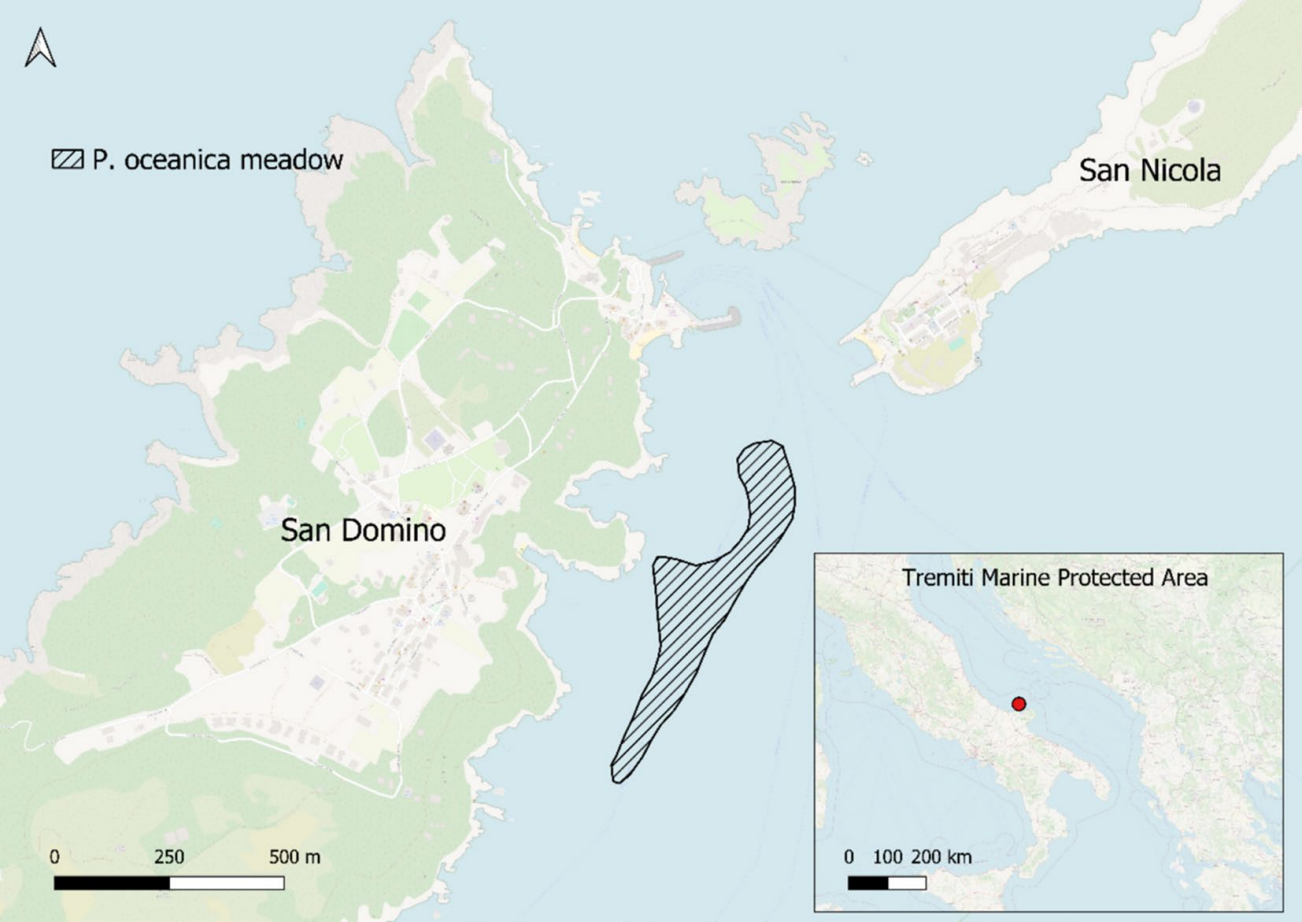
Graphical representation of the *P. oceanica* meadow selected for the case study in the Tremiti Islands marine protected area. The meadow, located off the coast of San Domino Island in the Tremiti Archipelago, evidenced by a striped pattern. Its shapefile was extracted from the dataset of Telesca et al. (2015). The inset map in the lower right corner shows the location of the marine protected area, in Southern Italy

Supported by the theoretical basis provided by the simulation study presented in the “Simulation study” and “Results from the simulation study” sections, this section empirically tests one of the proposed sampling schemes using real data from a meadow located in southern Italy. The selected *P. oceanica* meadow is situated in the Tremiti Islands Marine Protected Area, off the east coast of San Domino Island, Puglia Region (see Fig. 9). We used QGIS (QGIS.org., 2024) and R (R Core Team, 2023) for the empirical analysis.

The shapefile of the meadow was extracted from the Mediterranean seagrass map (Telesca et al., 2015, last accessed in April 2024). The meadow covers an area of 7.75 hectares. We used data from Tursi et al., 2022, who collected a non-probabilistic sample consisting of $R=27$ locations within the three sampling stations purposively positioned in the meadow (9 replicates from each station) in 2015 and another sample of the same size in 2020. The authors used a sample frame of 0.16 m^2^, recording, among others, the shoot density at each location.

To show the proposed estimation procedure, we assumed instead that the data were collected according to URS. Thus, we randomly generated 27 locations within the meadow, and we also assumed that these locations remained the same between 2015 and 2020. Then, we associated the generated locations with the data collected by Tursi et al. (2022) and computed the meadow’s density estimates in 2015 and 2020. We used this sample for the meadow’s density estimation, mapping, and monitoring. For monitoring purposes, we computed the estimate of density change based on Eq. (13).

For mapping purposes, we adopted the NN and the IDW interpolators (10) and (11) to provide the density maps for the years 2015 and 2020, providing also the maps of bootstrap estimates of precision achieved by (12). To achieve a satisfying map resolution, we constructed maps by estimating densities at the nodes of a 5 × 5 m grid. Following Di Biase et al. (2022), we performed IDW interpolation choosing the smoothing parameter $\alpha$ by means of a data-driven procedure of leave-one-out cross-validation fully described in that paper. For constructing the maps of precision, we used the interpolated maps as pseudo-populations, selecting $B=1000$ bootstrap samples of $R=27$ locations using the URS scheme that was assumed to be adopted for selecting the original sample. Then, for each node of the grid, we computed RMSEs according to Eq. (12).

### Results from the case study

Table 3 shows the estimation results for the meadow selected for the empirical analysis. In line with results from Tursi et al., 2022, we find that the change estimate ${\widehat{\Delta }}_{D,27}$ is negative, indicating that the shoot density in the *P. oceanica* meadow decreased from 2015 to 2020 by about 15 shoots/m^2^. This trend also aligns with previous findings in the literature (e.g., Marbà et al., 2014), which highlight a general decline of this habitat across the Mediterranean Sea. Moreover, some studies attribute this loss to anthropogenic factors such as anchoring and pollution (Hinz et al., 2024; Pergent-Martini et al., 2022). However, the confidence interval for this estimate includes zero, suggesting that the change is not statistically significant. To confirm this, a paired *t*-test was performed, which further indicated that the change in shoot density was not significant. Moreover, when compared to the average density of shoots/m^2^ obtained from ISPRA monitoring data collected within the Marine Strategy Framework Directive, i.e., 250 shoots/m^2^, the estimated density of the Tremiti Island meadow is significantly lower. This suggests that there is uncertainty in density estimates and conclusive evidence are still missing.

**Table 3:** Density estimates and density change estimates between 2015 and 2020 in the *P. oceanica* meadow selected for the case study in the Tremiti Islands marine protected area, presuming a URS of locations

Description	Estimate	2015	2020
Density	${\widehat{\overline{D}}}_{(27)}$	144.79	129.17
Variance	${v}_{27}$	6.50	11.62
95% confidence intervals	$0.95 C.I.$	131.78–157.80	105.92–152.40
Density change	${\widehat{\Delta }}_{D,27}$	− 15.63	
Variance	${v}_{27}$	11.29	
95% confidence intervals	$0.95 C.I.$	− 38.22–6.97	

**Figure 10:**
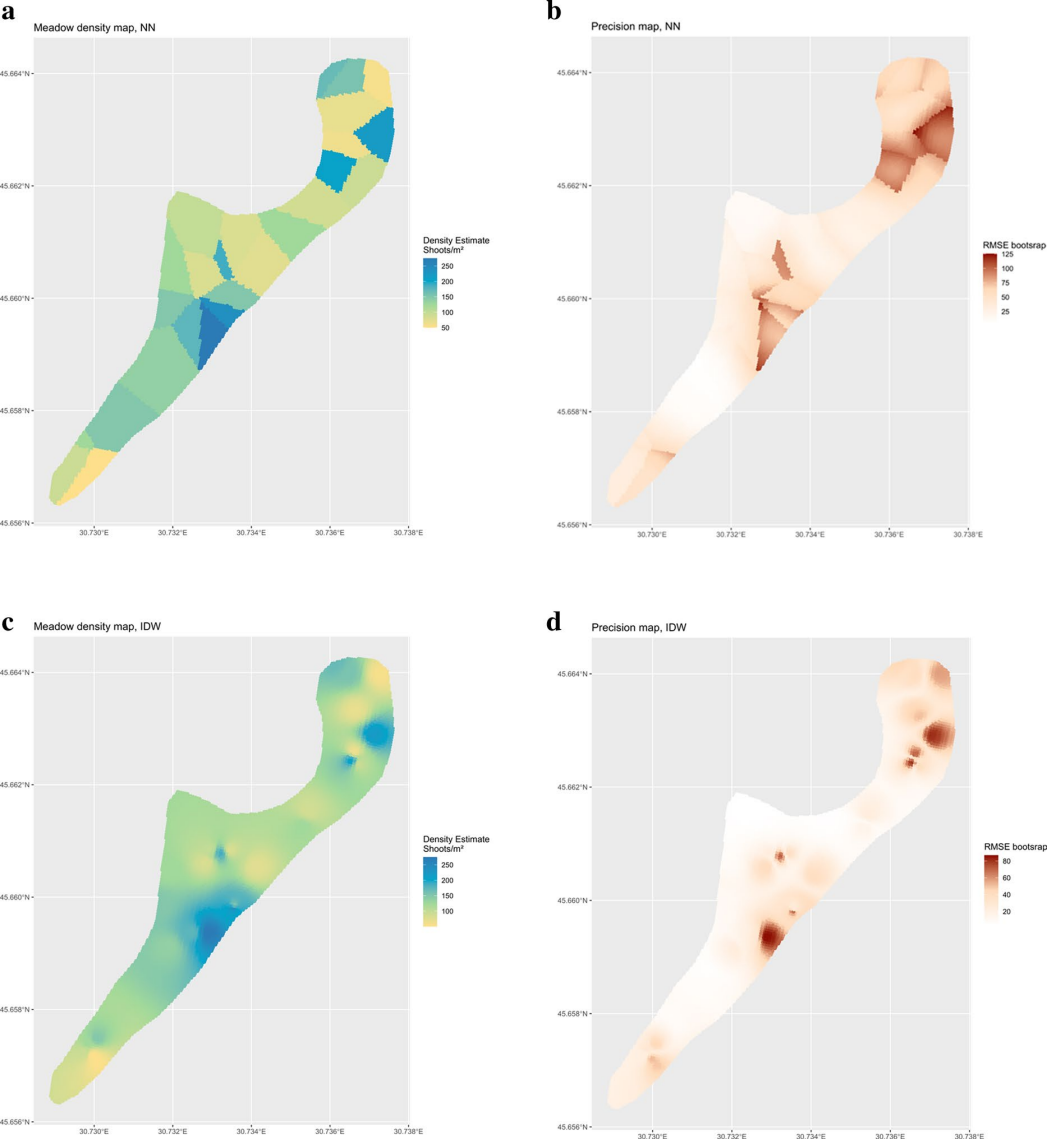
**a** Interpolated map of density achieved by means of NN interpolation of 27 density values recorded at locations supposed to be selected by URS. Blue areas indicate a high value of shoot density (number of shoots/m2), while light green and yellow colours represent lower density. **b** Bootstrap root mean squared error map from NN interpolation. **c** Interpolated map of density achieved by means of IDW interpolation of 27 density values recorded at locations supposed to be selected by URS. Blue areas indicate a high value of shoot density (number of shoots/m2), while light green and yellow colours represent lower density. **d** Bootstrap root mean squared error map from IDW interpolation

Figure 10a, b, c, and d shows the resulting interpolated maps and the corresponding precision maps for the two interpolation strategies (NN and IDW). The blue colour indicates higher values of density (shoot/m^2^), while light green/yellow areas are those in which the density is lower. For the precision maps, the dark red colour indicates higher values of RMSE, meaning a lower precision of interpolation. The density map obtained by using the IDW interpolator turns out to be, as expected, smoother. The IDW bootstrap root mean squared error map shows lower values, indicating that the interpolation by means of IDW is estimated to be more precise compared to the NN method.

**Figure 11:**
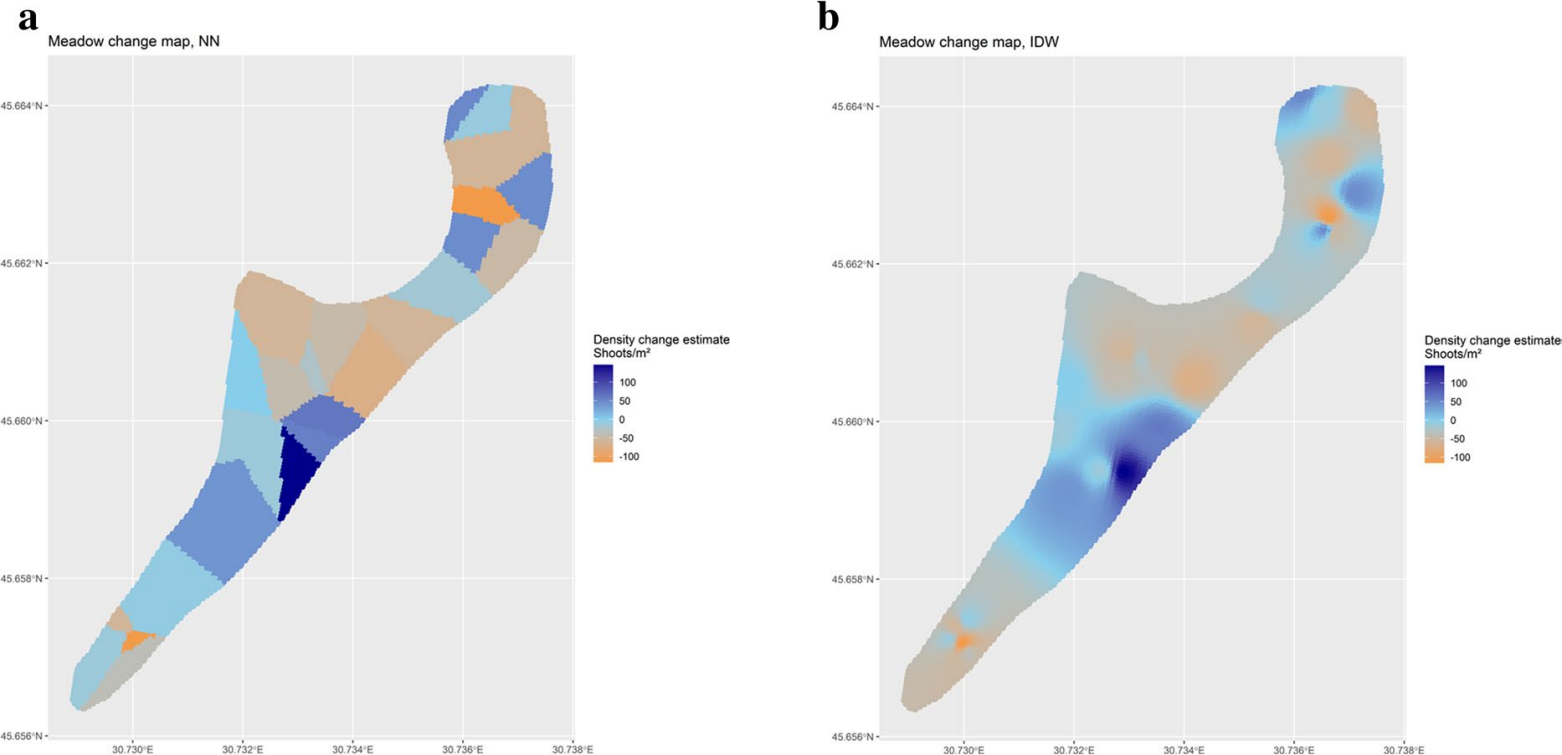
**a** Interpolated change density map by means of NN interpolator. Blue colour indicates that the shoot density (number of shoots/m^2^) increased from 2015 to 2020, while orange areas are characterised by a density decrease in the same period. **b** Interpolated change density map by means of IDW interpolator. Blue colour indicates that the shoot density (number of shoots/m^2^) increased from 2015 to 2020, while orange areas are characterised by a density decrease in the same period

The same procedure was applied to interpolate the map of density change between 2015 and 2020. Figure 11a and b shows the maps of density changes achieved by NN and IDW interpolations. Areas where densities increase are represented in blue, while areas where densities decrease are represented in oranges. All density changes are expressed in number of shoots per m^2^.

This case study showed that even with limited sampling effort, an appropriate probabilistic sampling scheme enables us to make reliable inferences for ecological attributes while providing a measure of precision (see Table 3). Furthermore, by using two simple interpolation methods, it is possible to generate maps of these attributes (density, in our case). This approach results in a dataset that meets the needs of effectively collecting, organising and making biophysical data accessible for reporting purposes. It considers habitat conditions, which affect the ecosystem’s capacity to provide ecosystem services and their consequent economic value, while also addressing the importance of managing uncertainty (Harwell et al., 2024). When comparing the results obtained by Tursi et al. (2022) with those from this case study, we observe a similar outcome. However, the authors primarily focus on monitoring sampling stations, as do several other studies assessing the status of *P. oceanica* (see, for example, Bellissimo et al., 2020). In fact, although the data used are the same, the proposed probability sampling strategy allows for sample interpolation in order to produce informative maps for EA purposes, thereby extending the usability of the results to the reporting required under the EA framework. Existing literature shows how probabilistic sampling schemes are already applied to assess the extent of different ecosystems, both for monitoring (see, e.g., Alessi et al., 2023; Corona et al., 2020) and accounting purposes (see Corona et al., 2012).

## Conclusions

This work addresses two main gaps in the EA literature: (i) the lack of rigorous sampling strategies to account for the extent and conditions of marine ecosystems and (ii) the need to align the current marine habitat monitoring strategies with the SEEA EA requirements. The move towards mandatory reporting of ecosystems—currently required for terrestrial ecosystems and likely to extend to marine ecosystems in the future—highlights the importance of collecting solid biophysical information. Furthermore, examples of ecosystem accounts for marine environments are scarce in the literature. In this context, accounting for the ecosystem services provided by habitats such as *P. oceanica* is essential, as is implementing strategies to collect continuous and reliable data. To fill these gaps, we propose the use of design-based inference for estimating, mapping, and monitoring marine habitats’ key ecological attributes, claiming how the use of probabilistic sampling schemes can support more precise and informative ecosystem accounts.

The estimation of ecosystems’ ecological attributes, as well as the mapping of their spatial distribution and monitoring over time, are essential features for compiling ecosystem accounts and recording changes in ecosystems’ extent and condition. Such information can also support evidence-based policymaking by providing indications of whether and where intervention is needed for habitat conservation, thereby ensuring the continuous provision of ecosystem services.

Our case study results suggest that mapping *P. oceanica* density and its temporal changes can identify specific areas requiring intervention, such as restoration efforts. For example, in the maps shown in our case study results, these areas are clearly visible at the individual meadow level. However, similar considerations can be made at larger scales by adapting the sampling strategy and the subsequent mapping approach. If *P. oceanica* density mapping were conducted at a national scale, similar to forest inventory efforts in Italy (see Fattorini et al., 2006), endangered seagrass meadows could be identified, and targeted interventions -such as transplanting- could be implemented. This approach would also help assess the effectiveness of marine protected areas in conserving this habitat and guide the selection of new protected sites.

The use of probabilistic sampling schemes enables not only the provision of reliable inferences for ecosystems’ ecological attributes but also the provision of precision measures, which are often overlooked. The proposed methodology also facilitates the integration of current monitoring strategies with the scope of the SEEA EA while ensuring compliance with international directives, such as the EU Habitats Directive and the Marine Strategy Framework Directive.

While our primary focus is on *P. oceanica* shoot density, our proposal can be adapted to different ecological attributes and diverse habitats, offering a flexible framework for collecting and analysing biophysical data that complies with international directives. Moreover, while we focus on meadows of a few tens of hectares where a single-phase sampling scheme can be employed, it is crucial to notice that our methodology can be extended to larger scales. It can be applied to single larger meadows as well as at a national scale, using two-stage sampling schemes in which portions of large meadows or small meadows are selected in the first stage and the proposed sampling strategy is performed in the second stage within the units selected in the first one.

The benefits of appropriate sampling schemes for assessing *P. oceanica* are explored via simulation testing on four artificial populations. Simulation results show that even when the sampling effort is small relative to the meadow size, the selected sampling schemes, i.e., URS and TSS, perform well, with precision varying according to the spatial patterns of the populations. This ensures the practical feasibility of our approach. Finally, we demonstrate the empirical viability of our proposal using data collected on a meadow located in an Italian Marine Protected Area. The case study also demonstrates the methodology’s effectiveness in producing a dataset that provides reliable spatial information that can be used for EA and monitoring purposes.

## Supplementary Information

Below is the link to the electronic supplementary material.

Supplementary file1 (DOCX 15358 KB)

## Data Availability

Data and codes used for the simulation study and for the case study are available on request. Data on Posidonia oceanica meadows (Mediterranean seagrass map) are available in Telesca et al., 2015.

## Footnotes

1 Quadrat plots are the most common sampling devices used by divers to record *P. oceanica* shoot density (Bellissimo et al., 2020; Gullién et al., 2013; Lopez y Royo et al., 2010; Pergent-Martini et al., 2021; Ramos Esplá et al., 2005; Romero et al., 2007; Tursi et al., 2022).

2 The number of points was derived considering the average density obtained from ISPRA monitoring data and the size of the artificially generated grassland (35 hectares).
