# Supplementary material for: A probabilistic sampling strategy for estimating plant density in Posidonia oceanica meadows
Source: Environ Monit Assess. 2025 Apr 11;197(5):541. doi: 10.1007/s10661-025-13973-z (PMC11991980; doi:10.1007/s10661-025-13973-z)
Supplement: Supplementary file 1 — Supplementary file1 (DOCX 15358 KB) [file 10661_2025_13973_MOESM1_ESM.docx]

**PROBABILISTIC SAMPLING STRATEGY FOR ESTIMATING PLANT DENSITY IN POSIDONIA MEADOWS**

**Supplementary materials**

**Alice Bartolini^a^, Agnese Marcelli^b^, Rosa Maria Di Biase^b^, Lorenzo Fattorini^b^, Silvia Ferrini^b,c,d^**

^a^ University of Trento

^b^ University of Siena

^c^ University of East Anglia

^d^ University College of London

**Bias and root mean squared error (RMSE) maps**

For each population (regular, trended, clustered and striped patterns), sampling scheme (URS and TSS) and sample size ($n=25, 36, 49, 64)$, we estimate $M = 10 000$ density maps - one for each selected sample - according to the two selected interpolation methods: NN (9) and IDW (10). We then compare the resulting estimated density maps with the real maps and provide precision maps using the bias and root mean squared error (RMSE) precision indexes, described by equations (24) and (25), respectively.

The resulting maps are presented below. The IDW interpolator generally performs better than the NN interpolator across all populations. Additionally, increasing the sample size improves the precision of estimates. This is particularly evident when examining the bias and RMSE maps of population 2 (both for NN and IDW interpolations) and the IDW - RMSE maps of population 4. For population 1, characterised by a regular spatial pattern, good precision is achieved even with small sample sizes. In contrast, the results for population 3 indicate that the selected sample sizes are too low for such a clustered pattern, and no significant improvement is obtained when increasing it from $n=25$ to $n=64$.

| **Figure SM1**: Population 1 bias map, URS, NN interpolator |
| --- |
| 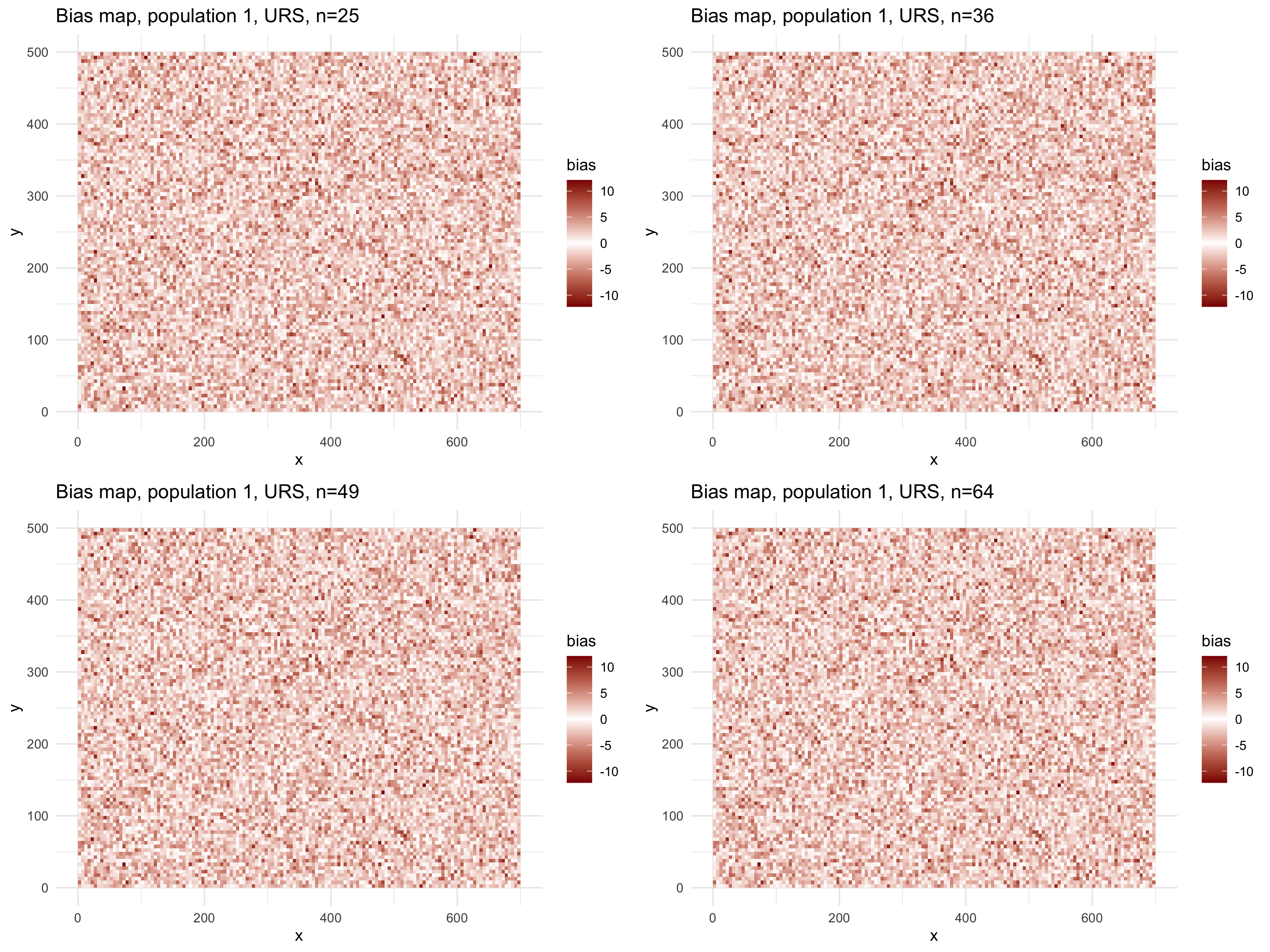 |
| **Figure SM2**: Population 1 RMSE map, URS, NN interpolator |
| 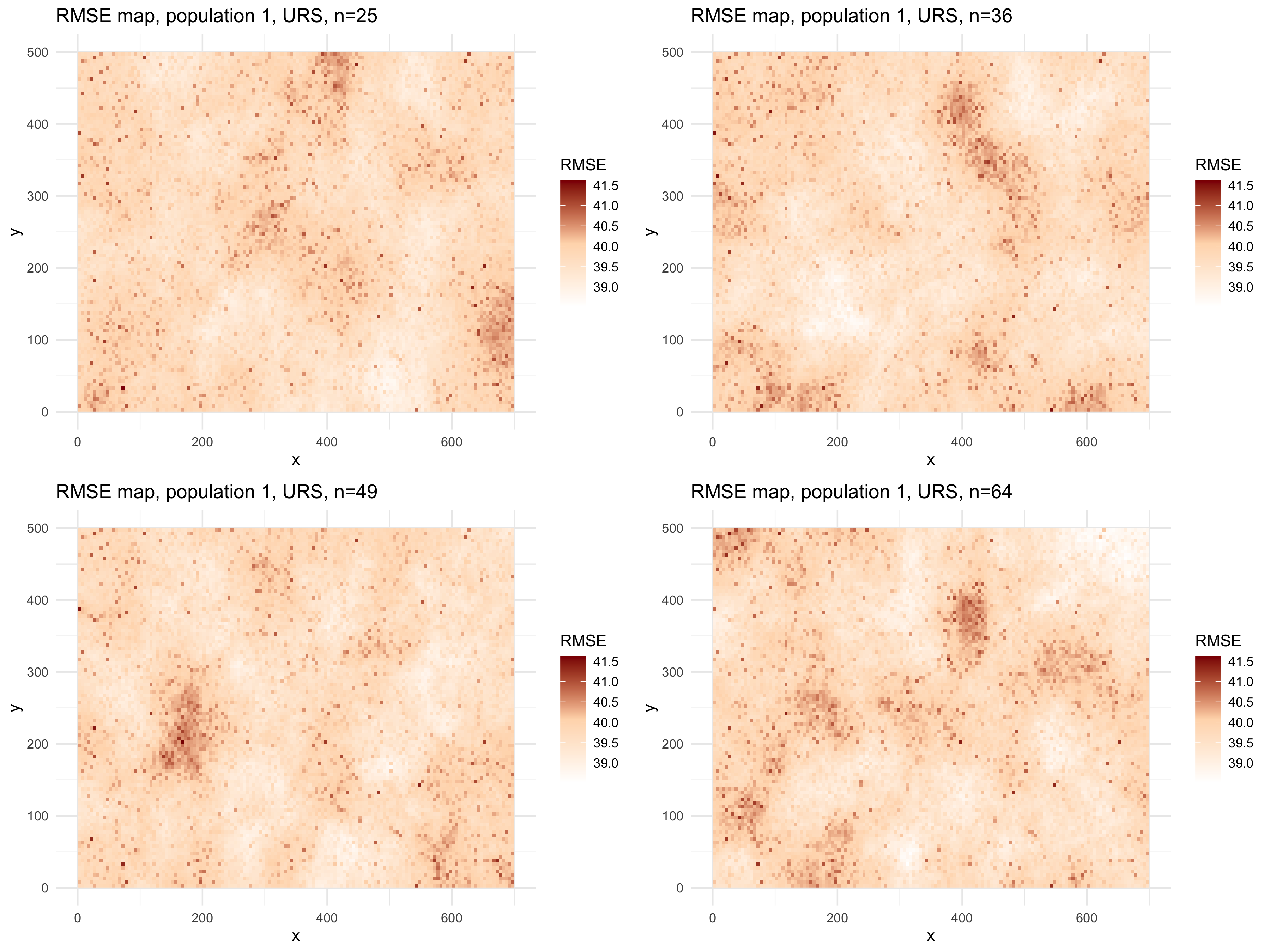 |

| **Figure SM3**: Population 1 bias map, TSS, NN interpolator |
| --- |
| 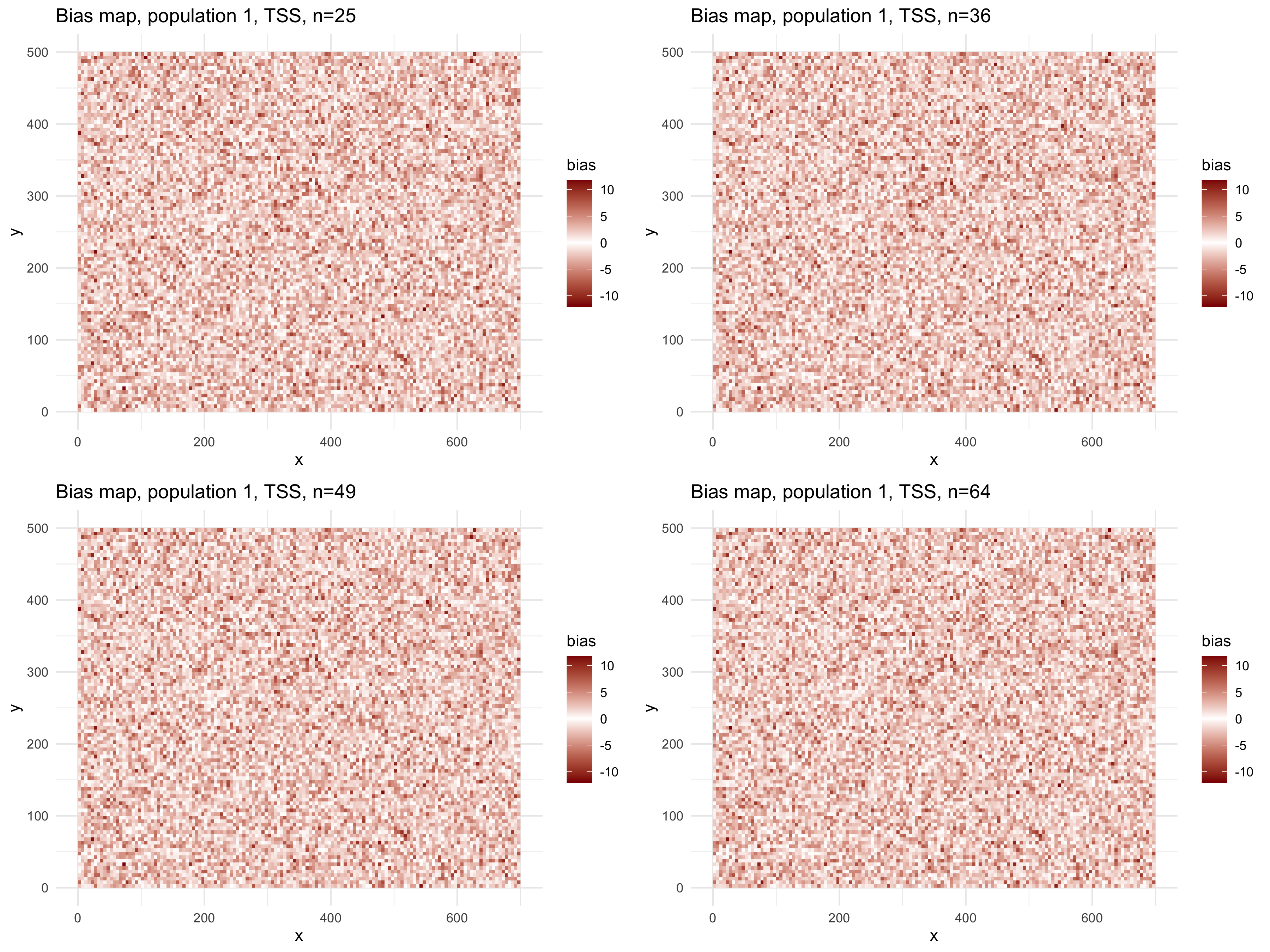 |
| **Figure SM4**: Population 1 RMSE map, TSS, NN interpolator |
| 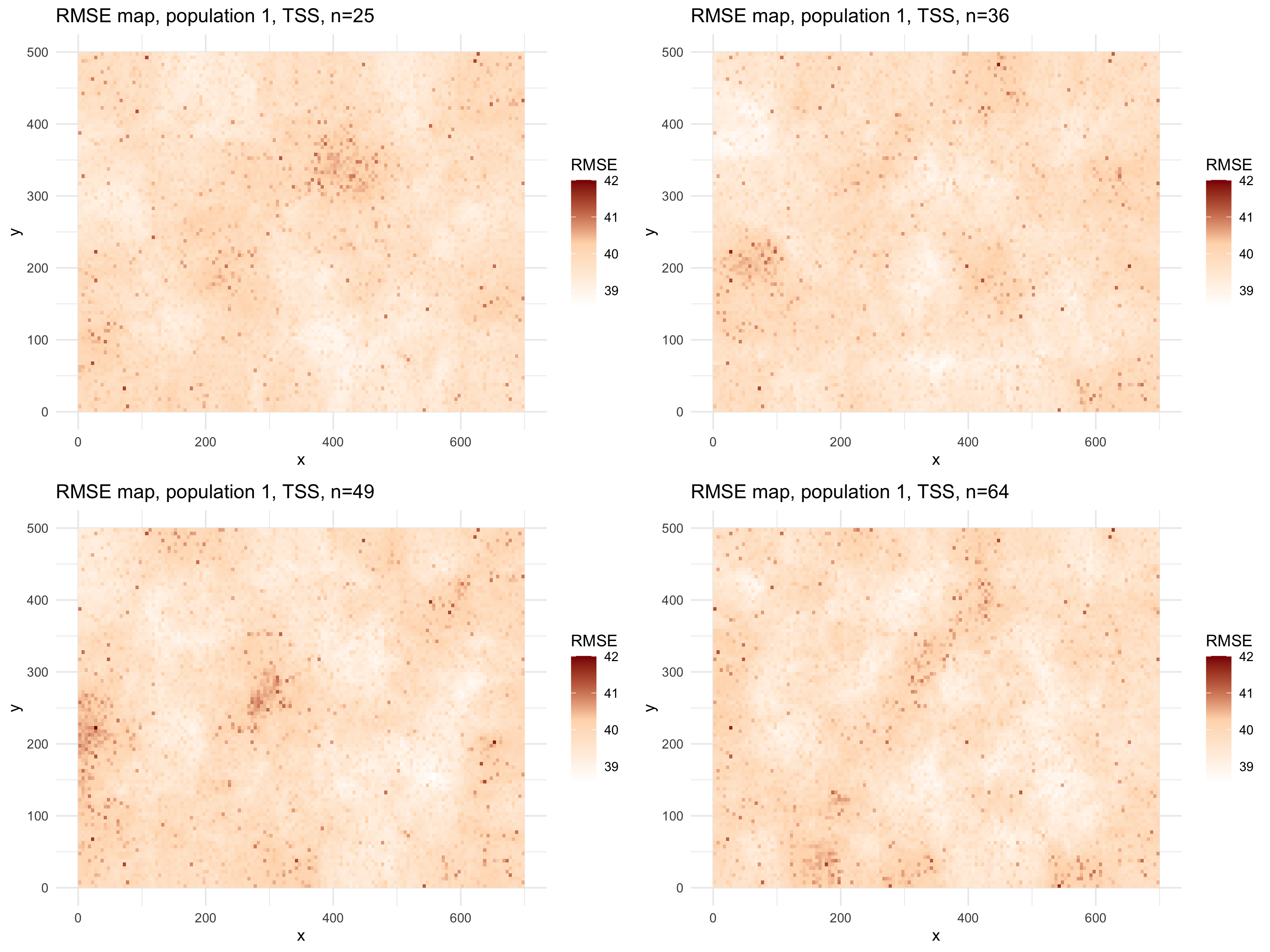 |

| **Figure SM5**: Population 1 bias map, URS, IDW interpolator |
| --- |
| 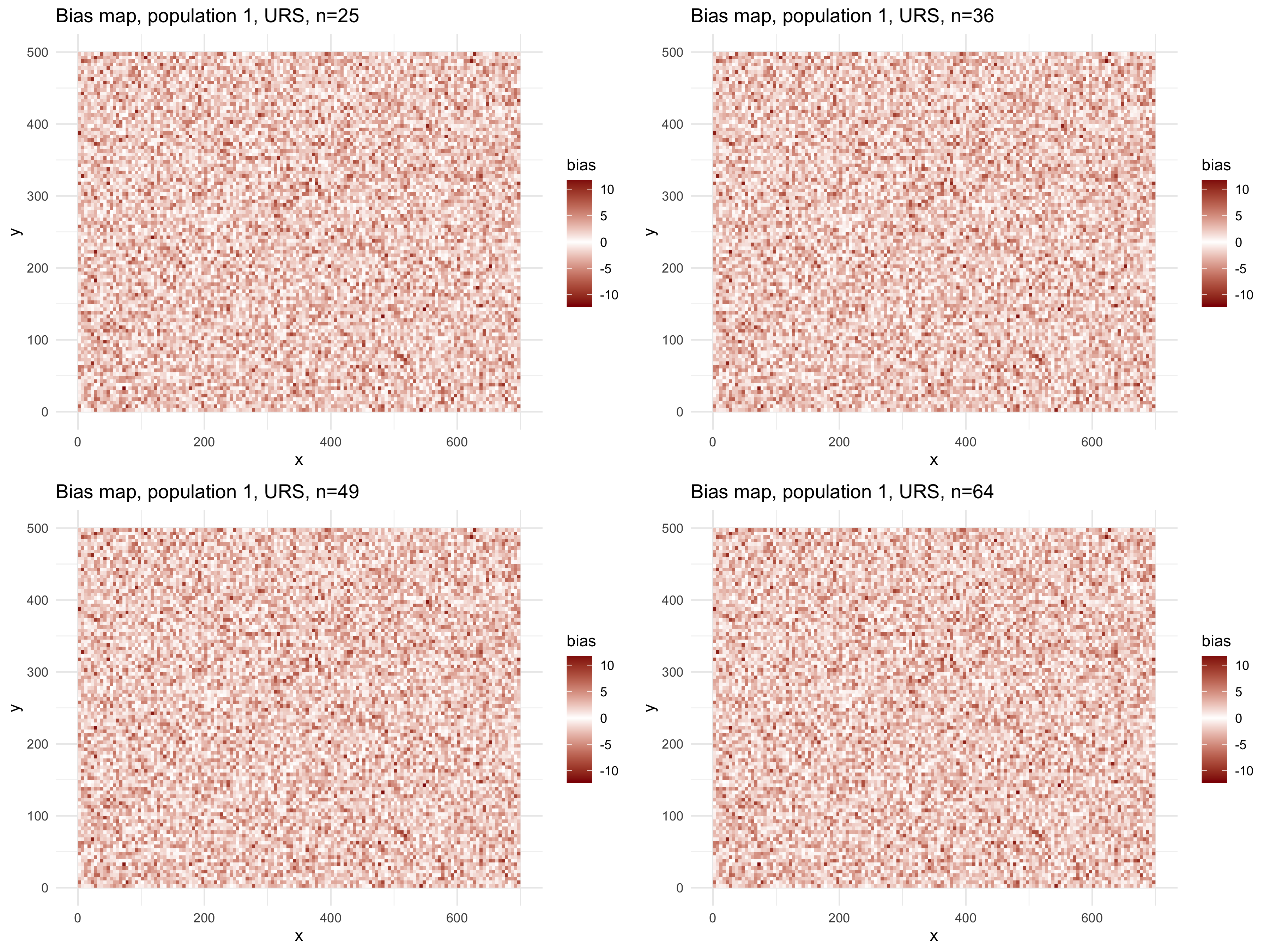 |
| **Figure SM6**: Population 1 RMSE map, URS, IDW interpolator |
| 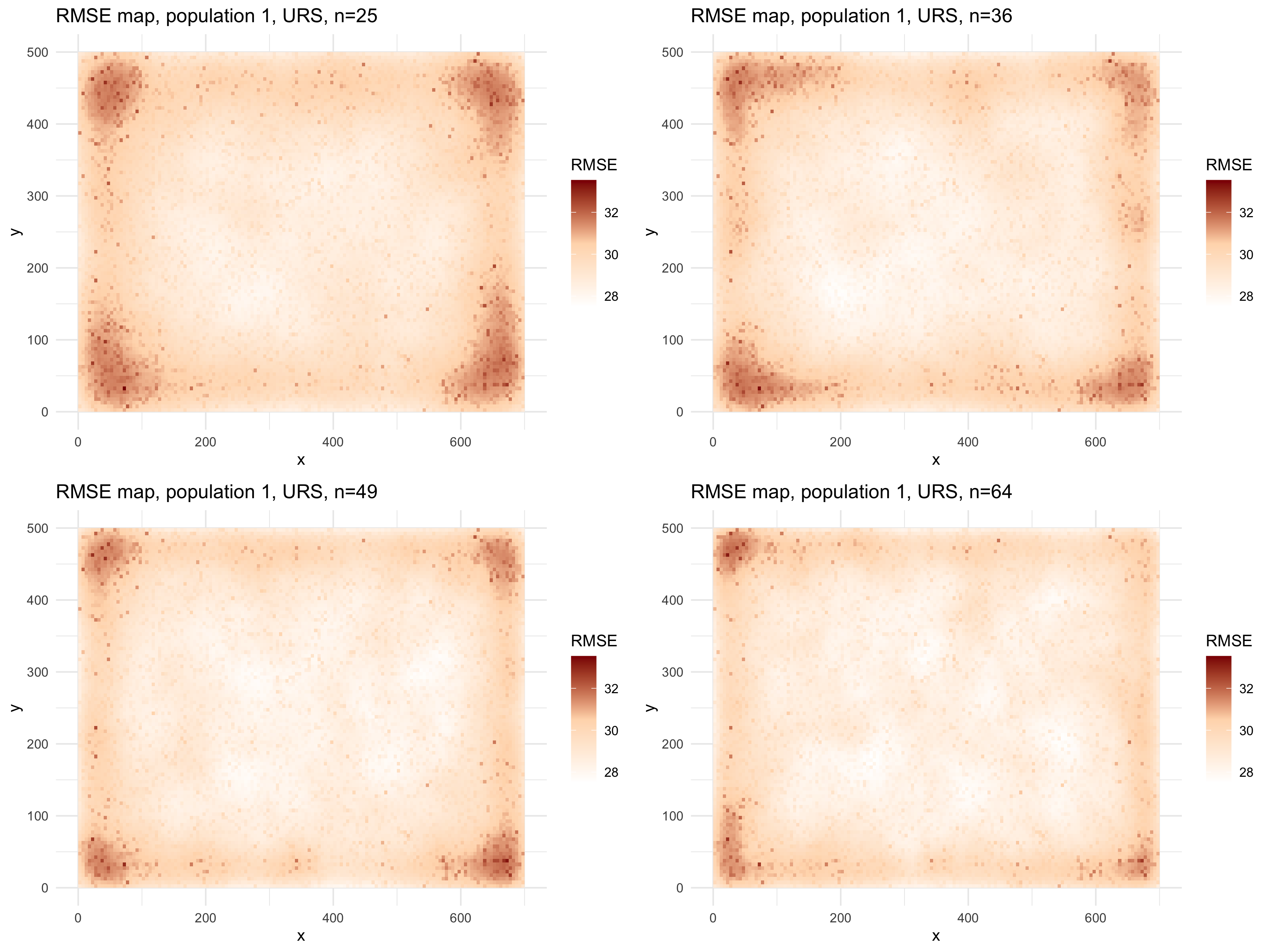 |

| **Figure SM7**: Population 1 bias map, TSS, IDW interpolator |
| --- |
| 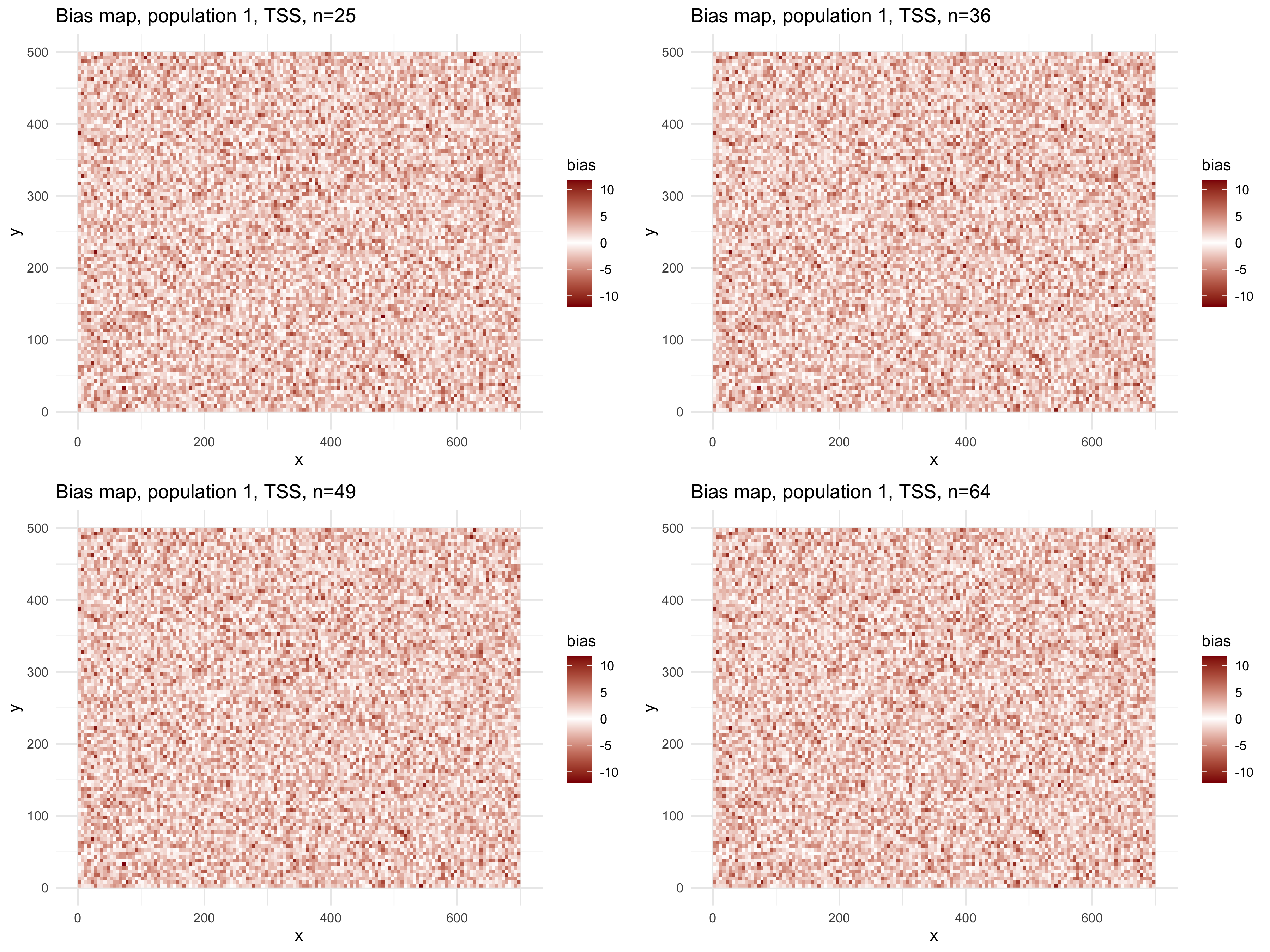 |
| **Figure SM8**: Population 1 RMSE map, TSS, IDW interpolator |
| 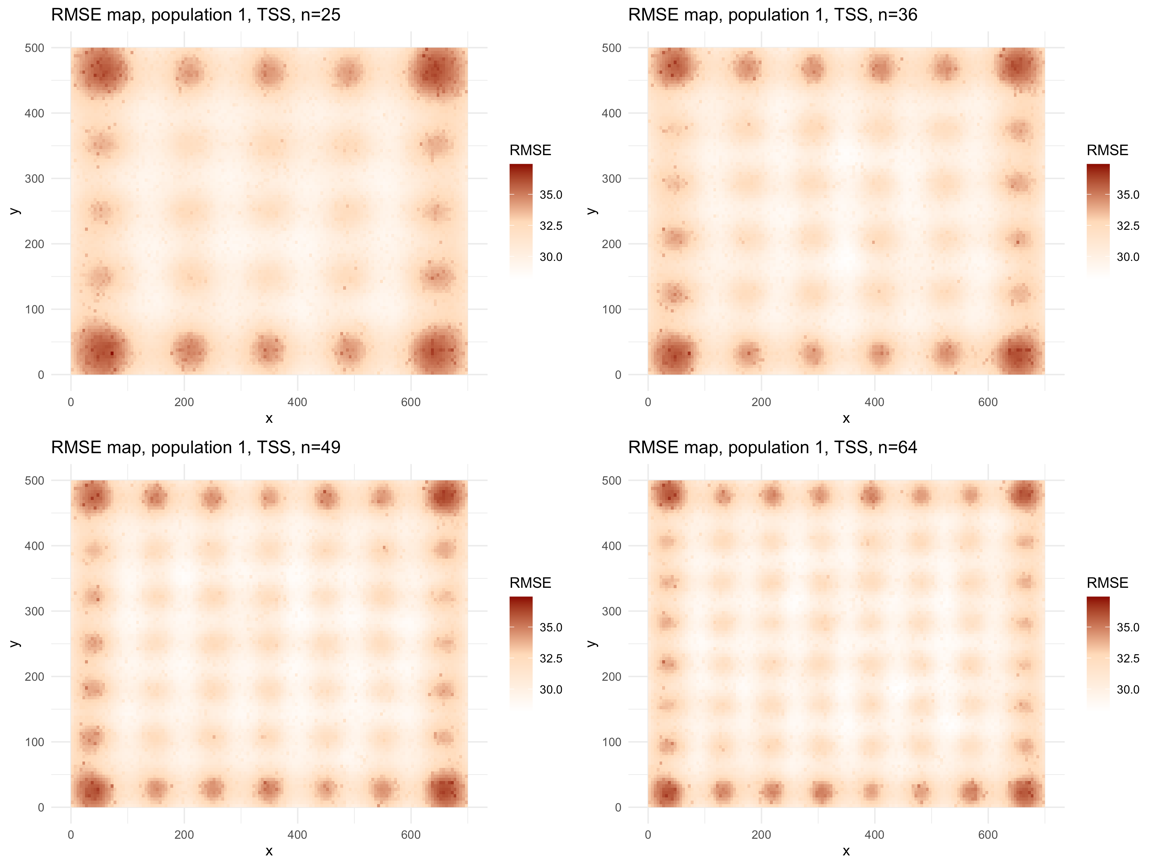 |

| **Figure SM9**: Population 2 bias map, URS, NN interpolator |
| --- |
| 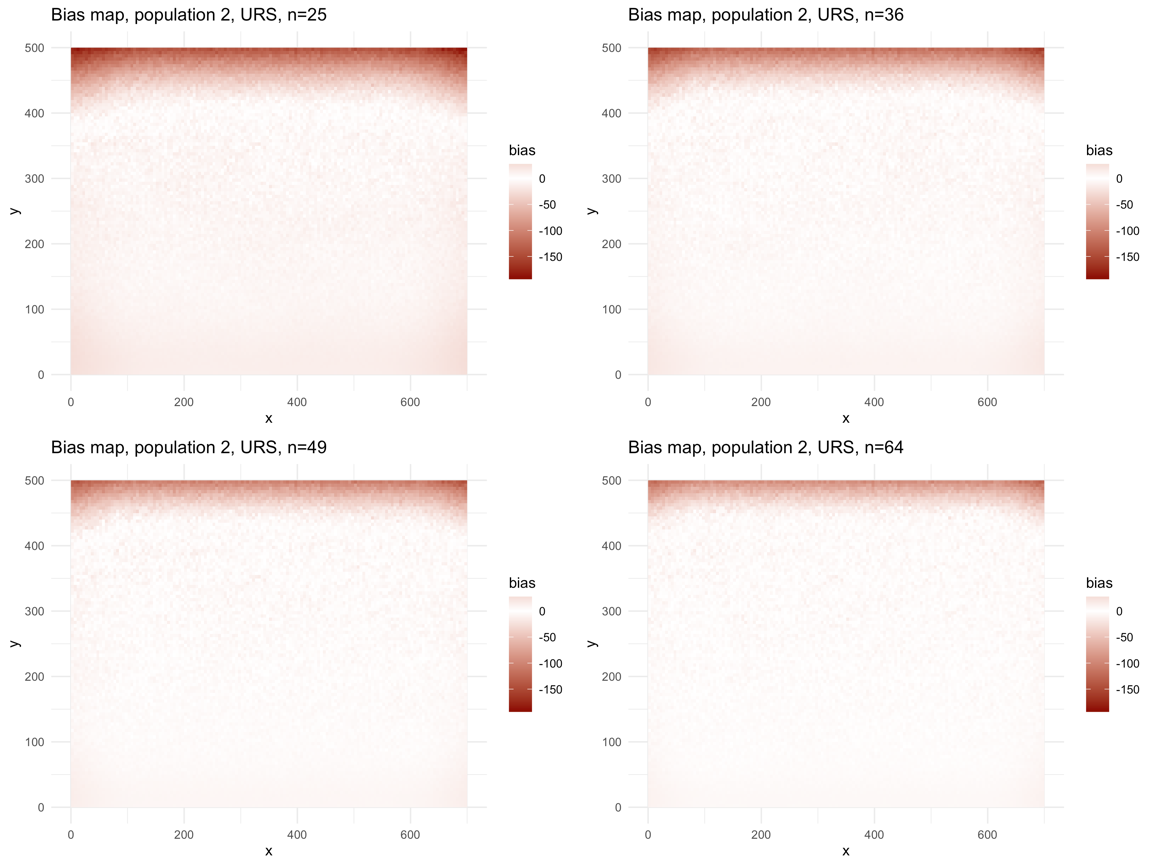 |
| **Figure SM10**: Population 2 RMSE map, URS, NN interpolator |
| 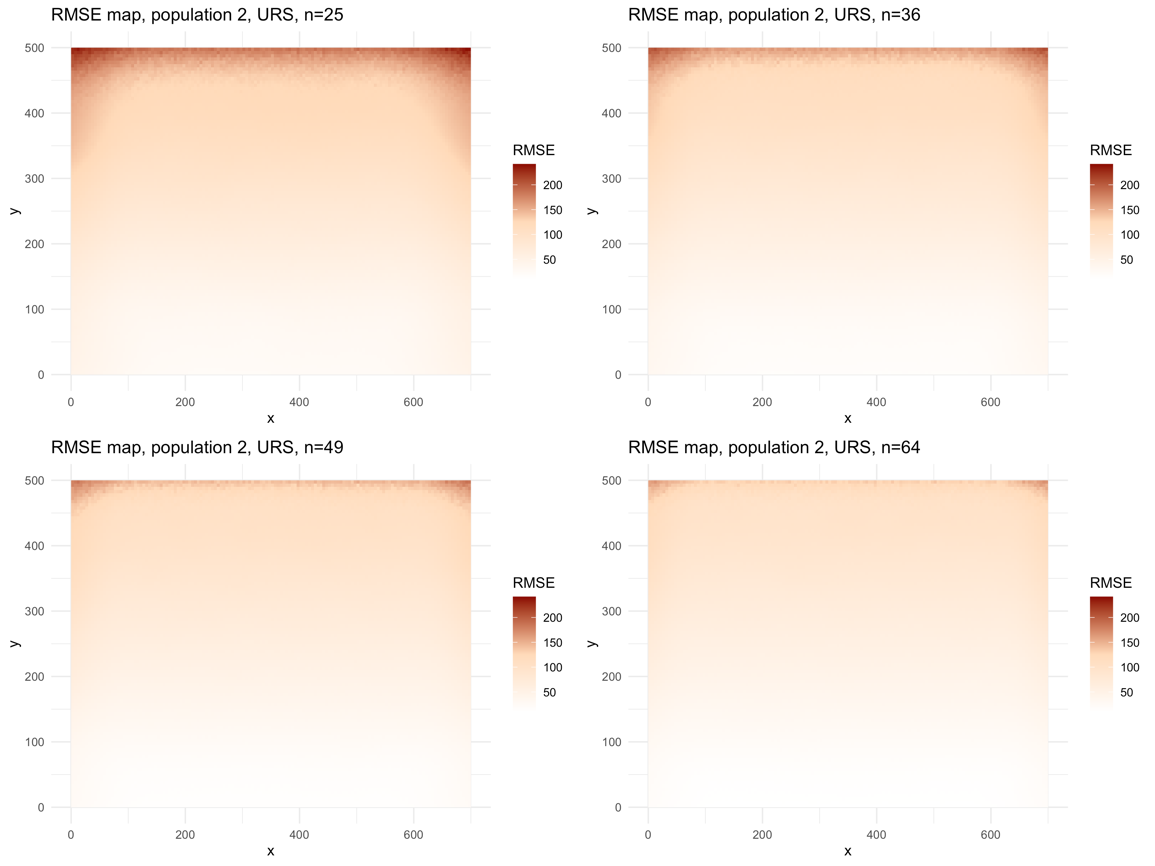 |

| **Figure SM11**: Population 2 bias map, TSS, NN interpolator |
| --- |
| 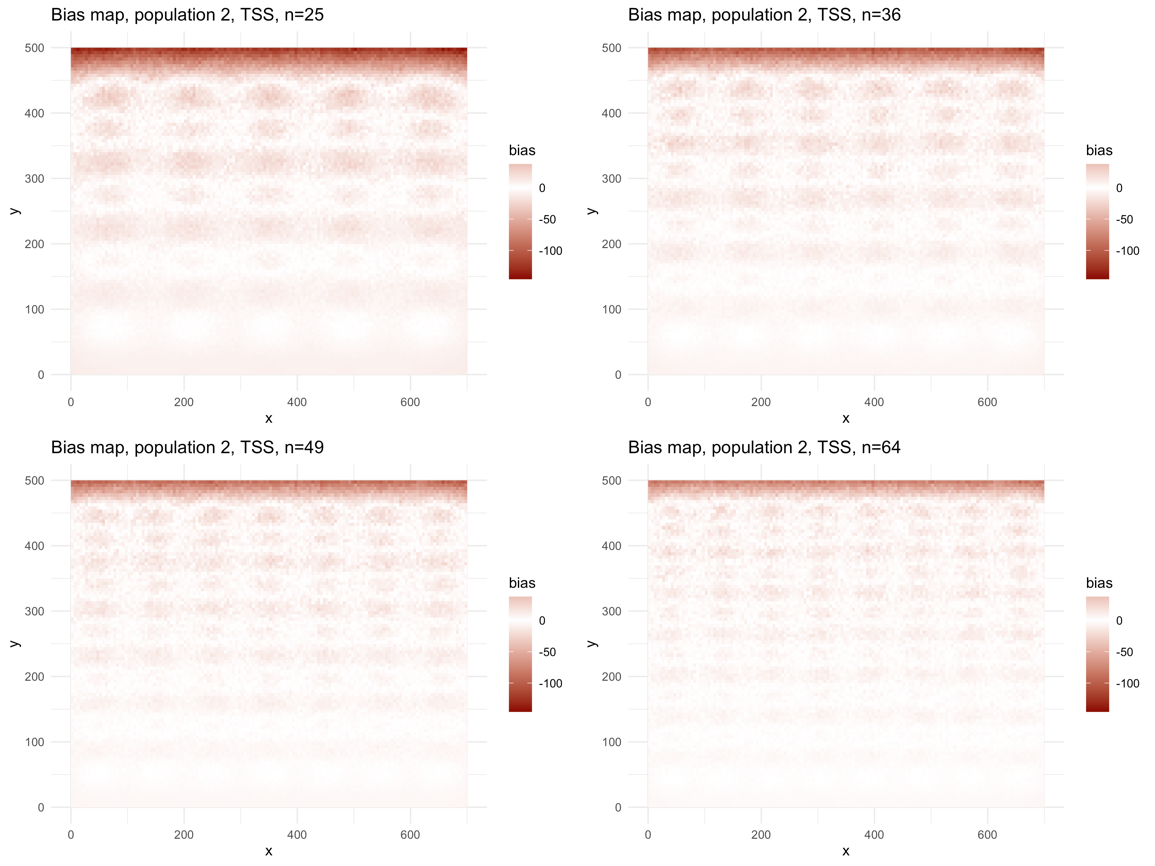 |
| **Figure SM12**: Population 2 RMSE map, TSS, NN interpolator |
| 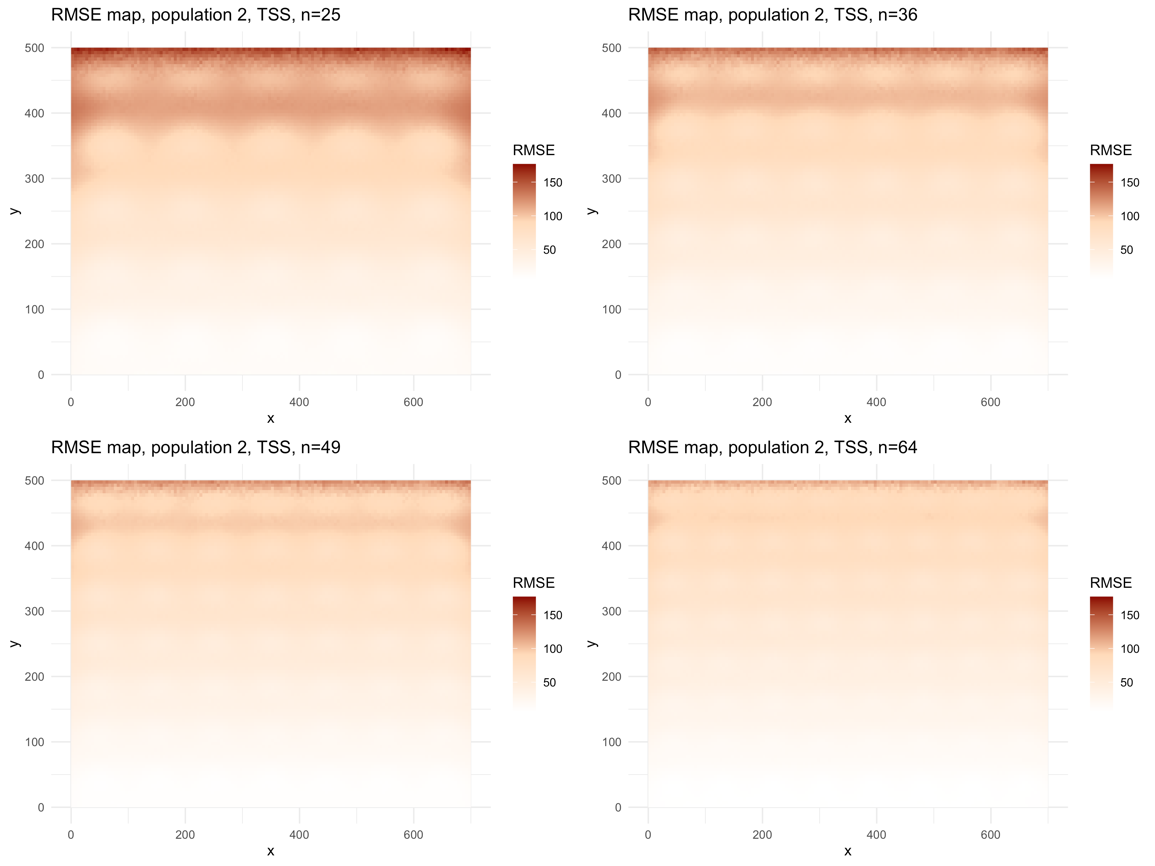 |

| **Figure SM13**: Population 2 bias map, URS, IDW interpolator |
| --- |
| 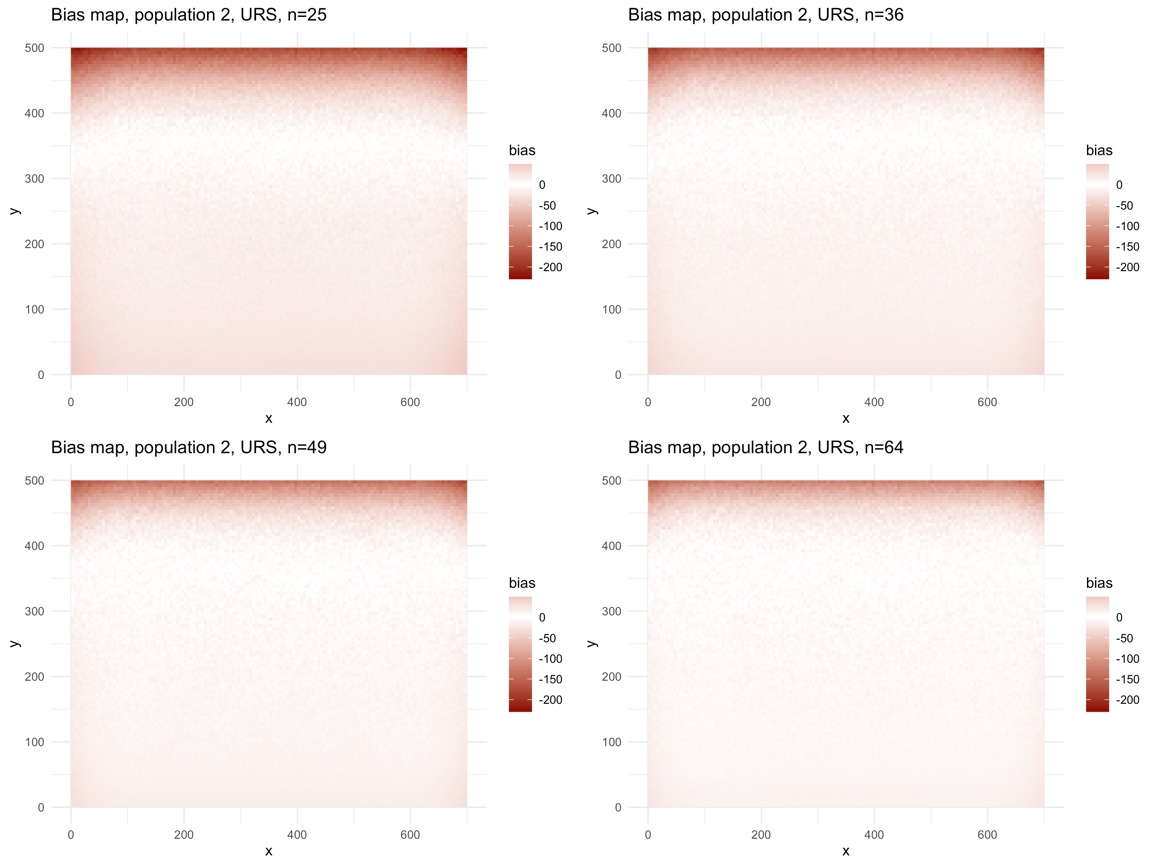 |
| **Figure SM14**: Population 2 RMSE map, URS, IDW interpolator |
| 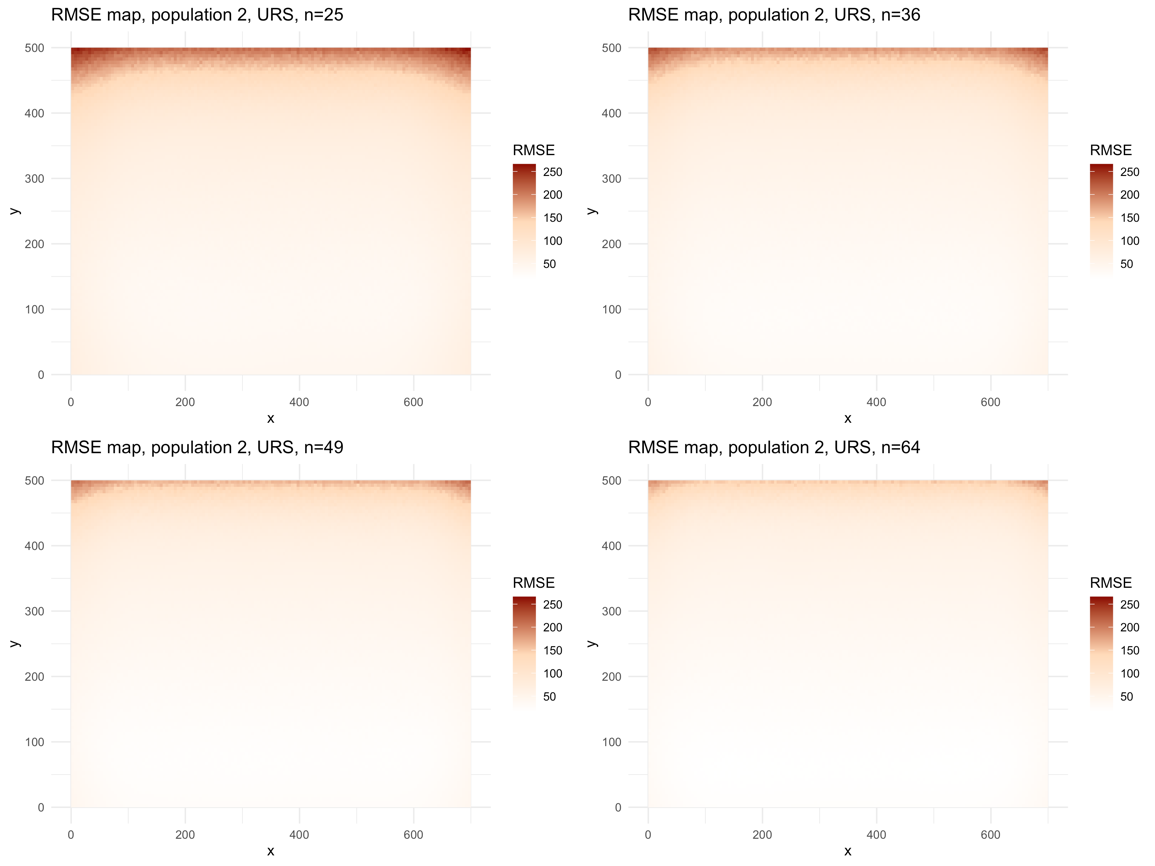 |

| **Figure SM15**: Population 2 bias map, TSS, IDW interpolator |
| --- |
| 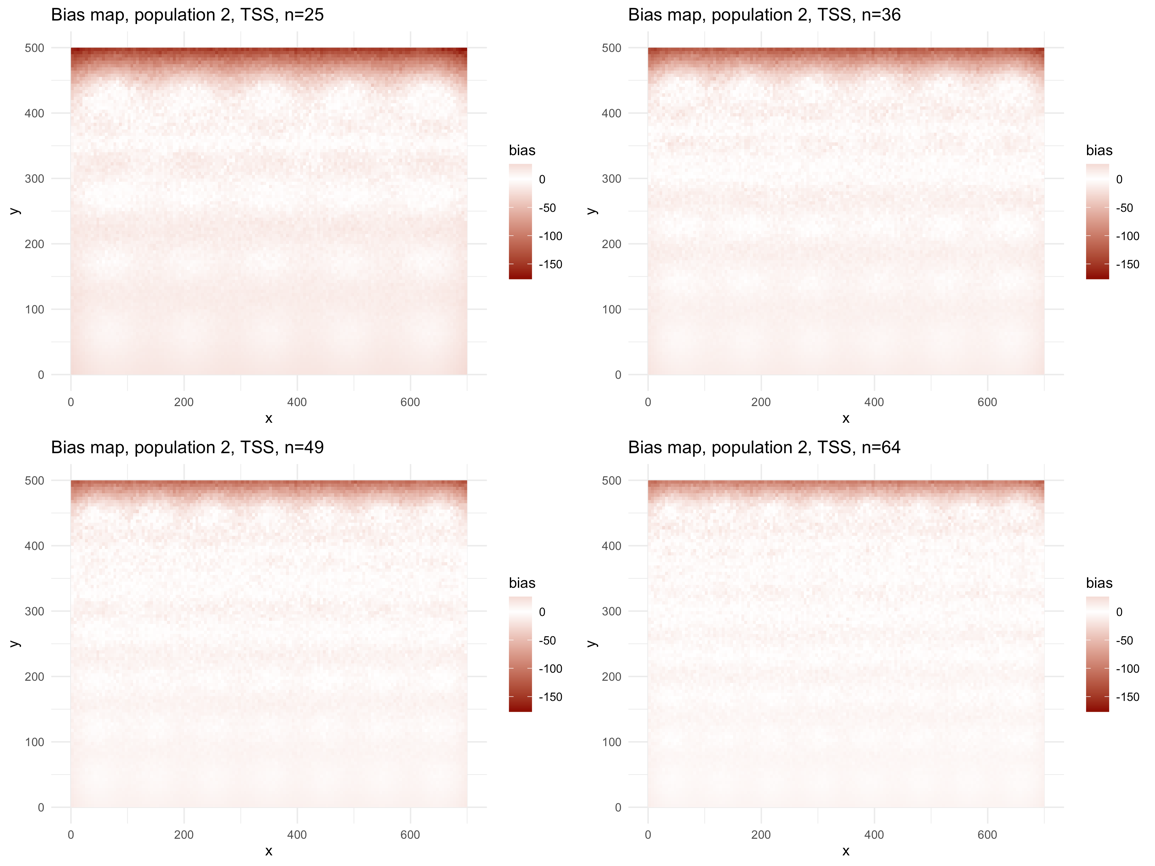 |
| **Figure SM16**: Population 2 RMSE map, TSS, IDW interpolator |
| 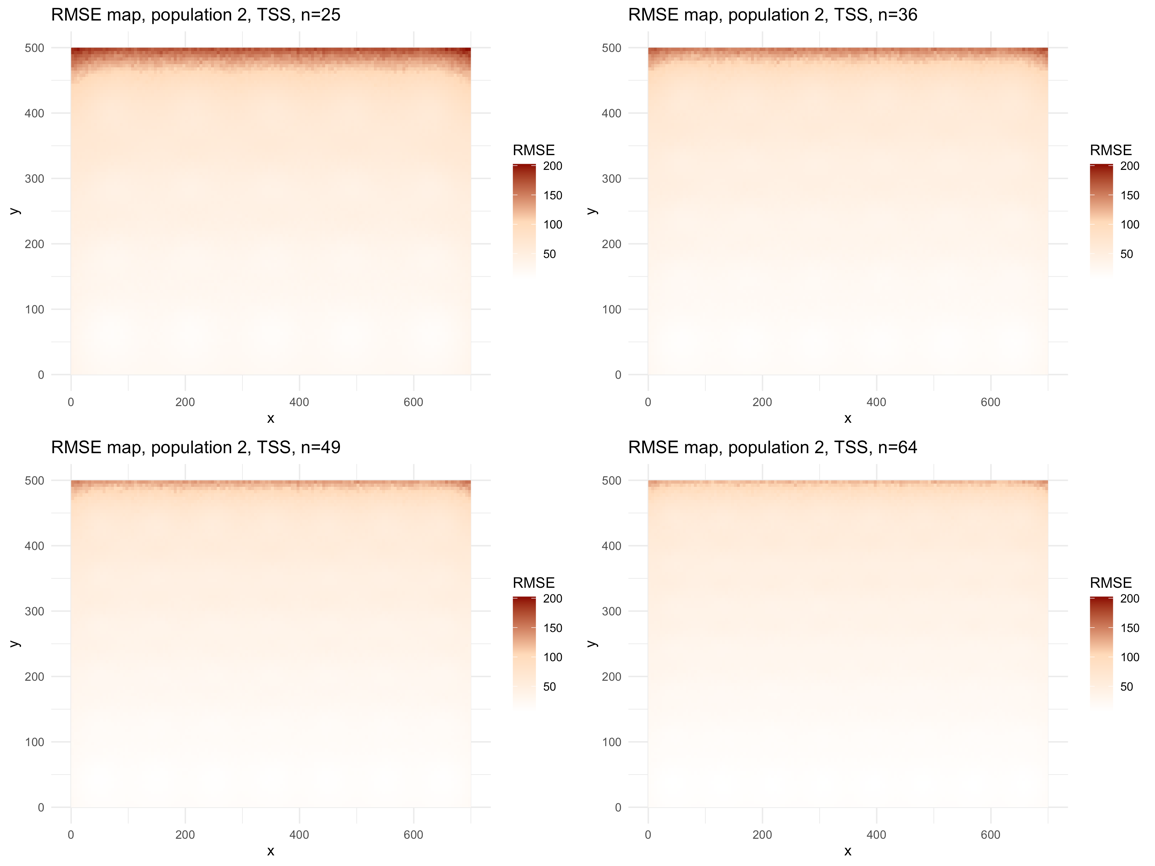 |

| **Figure SM17**: Population 3 bias map, URS, NN interpolator |
| --- |
| **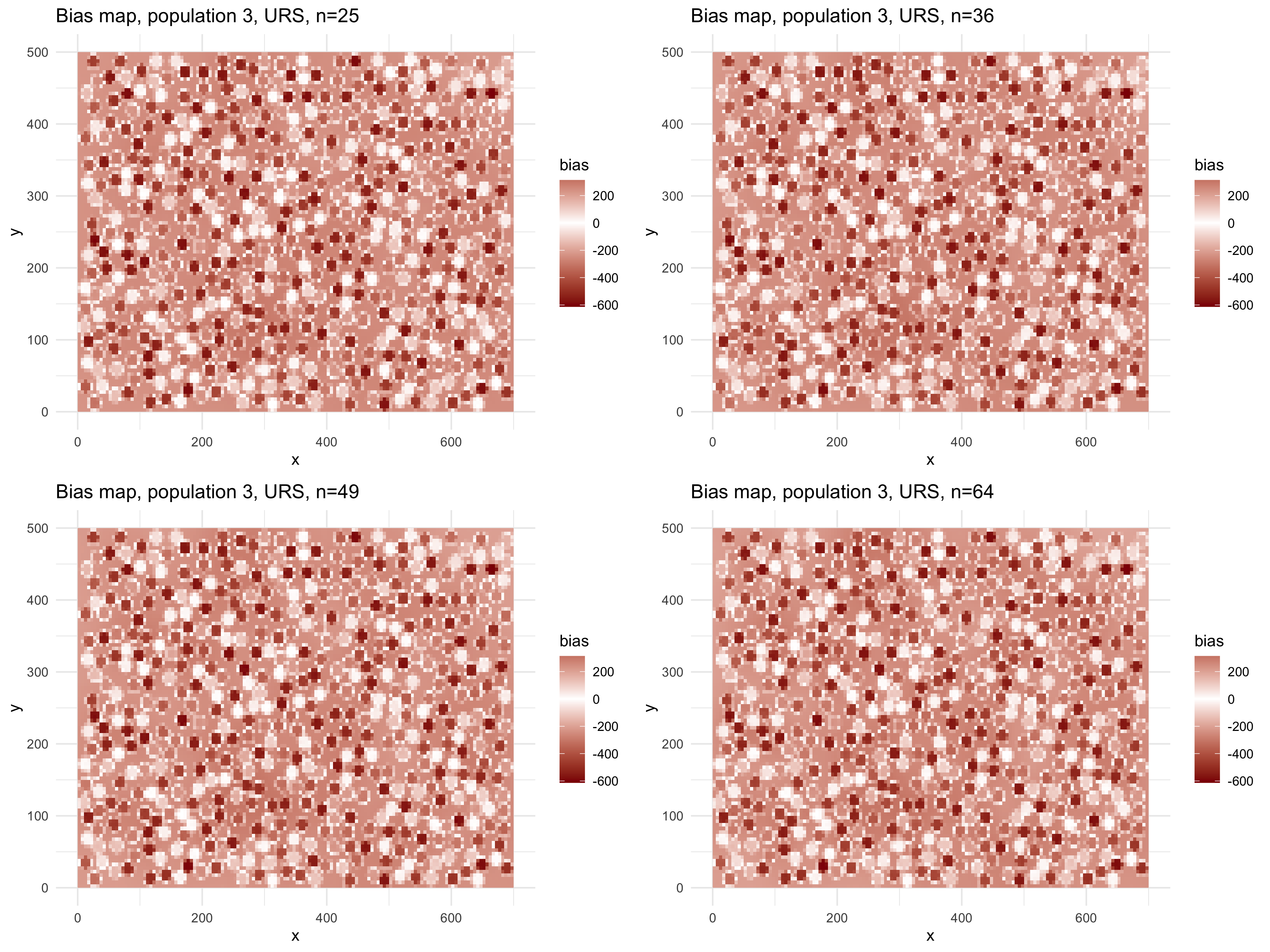** |
| **Figure SM18**: Population 3 RMSE map, URS, NN interpolator |
| **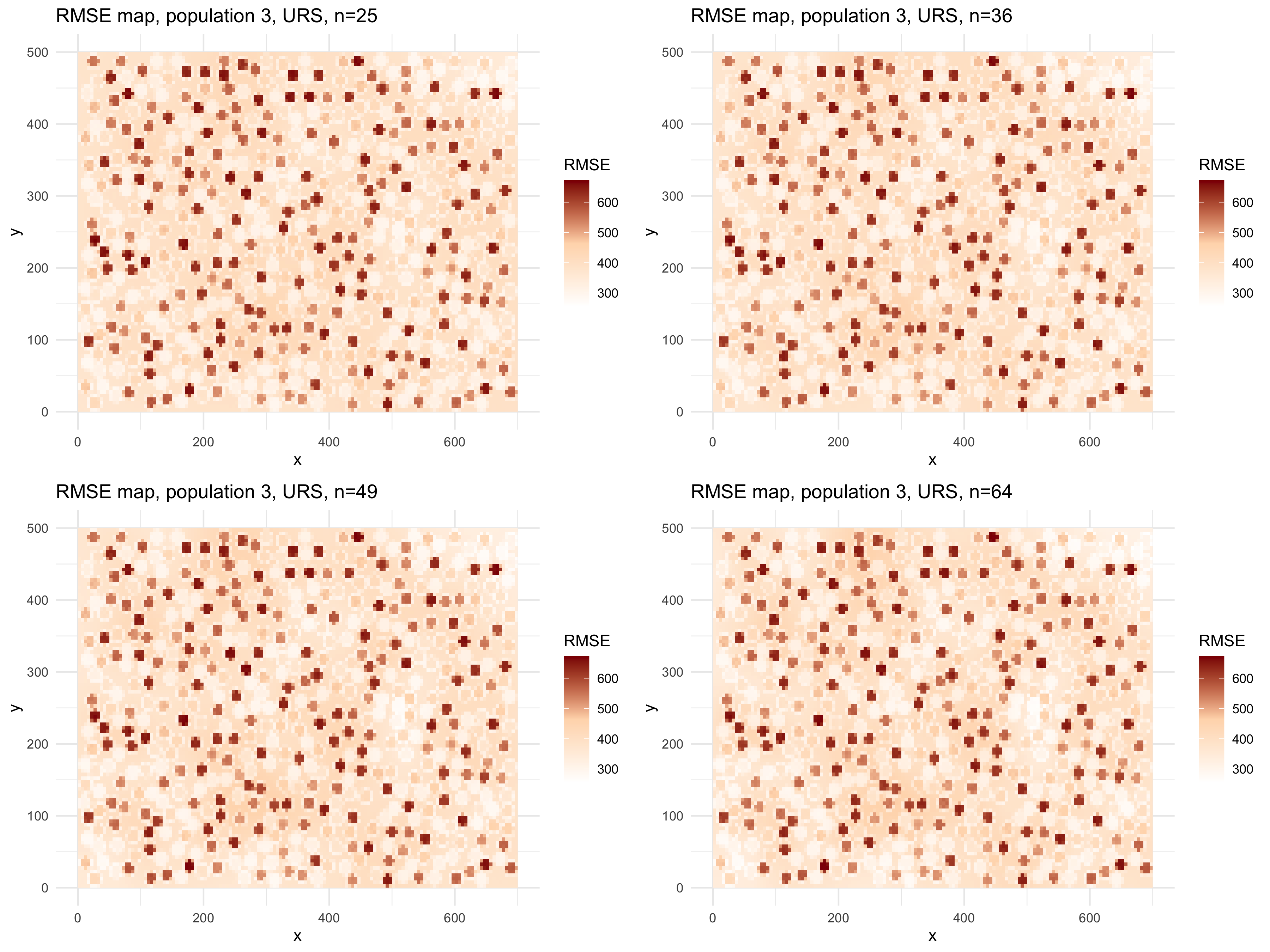** |

| **Figure SM19**: Population 3 bias map, TSS, NN interpolator |
| --- |
| 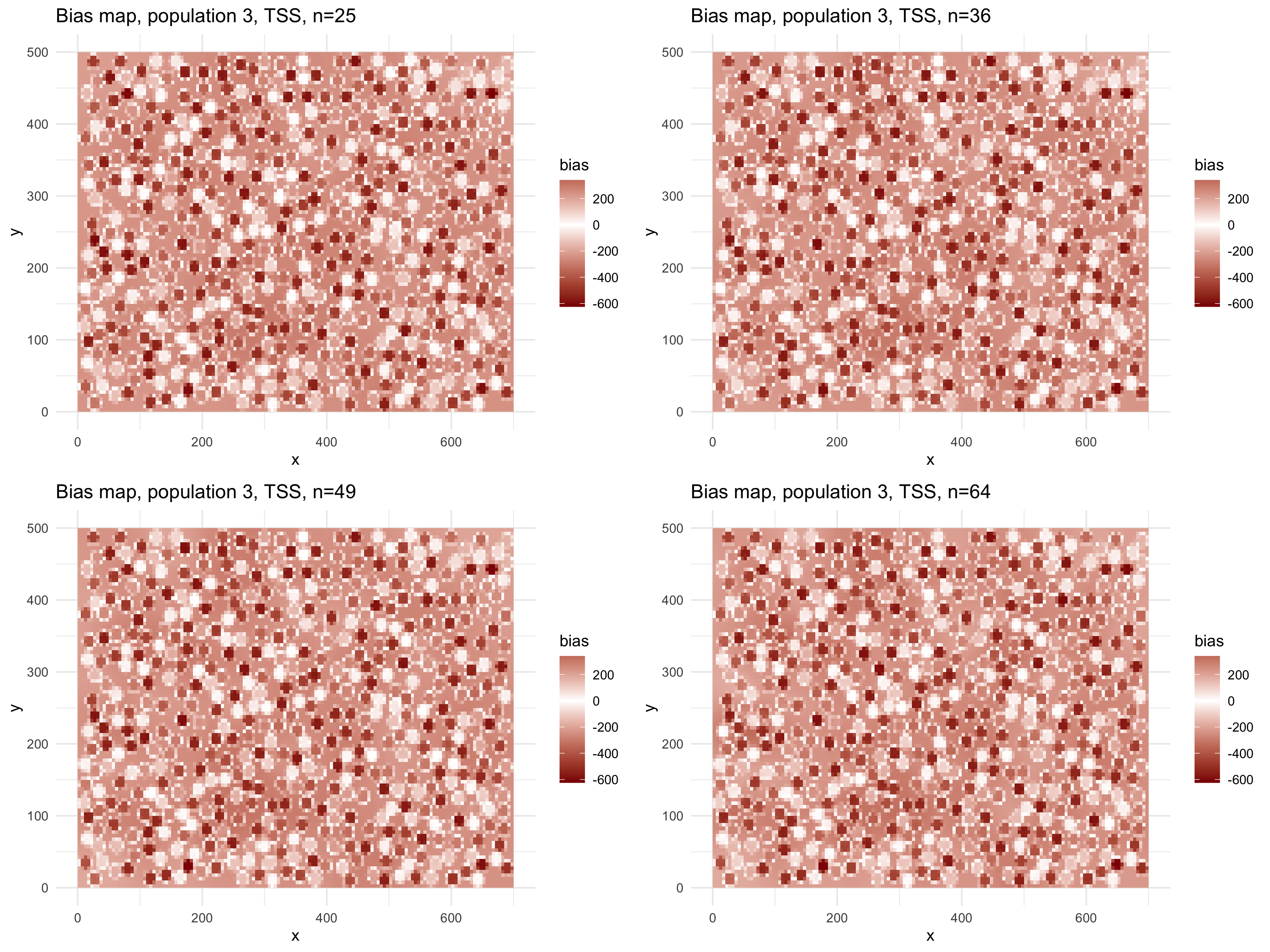 |
| **Figure SM20**: Population 3 RMSE map, TSS, NN interpolator |
| **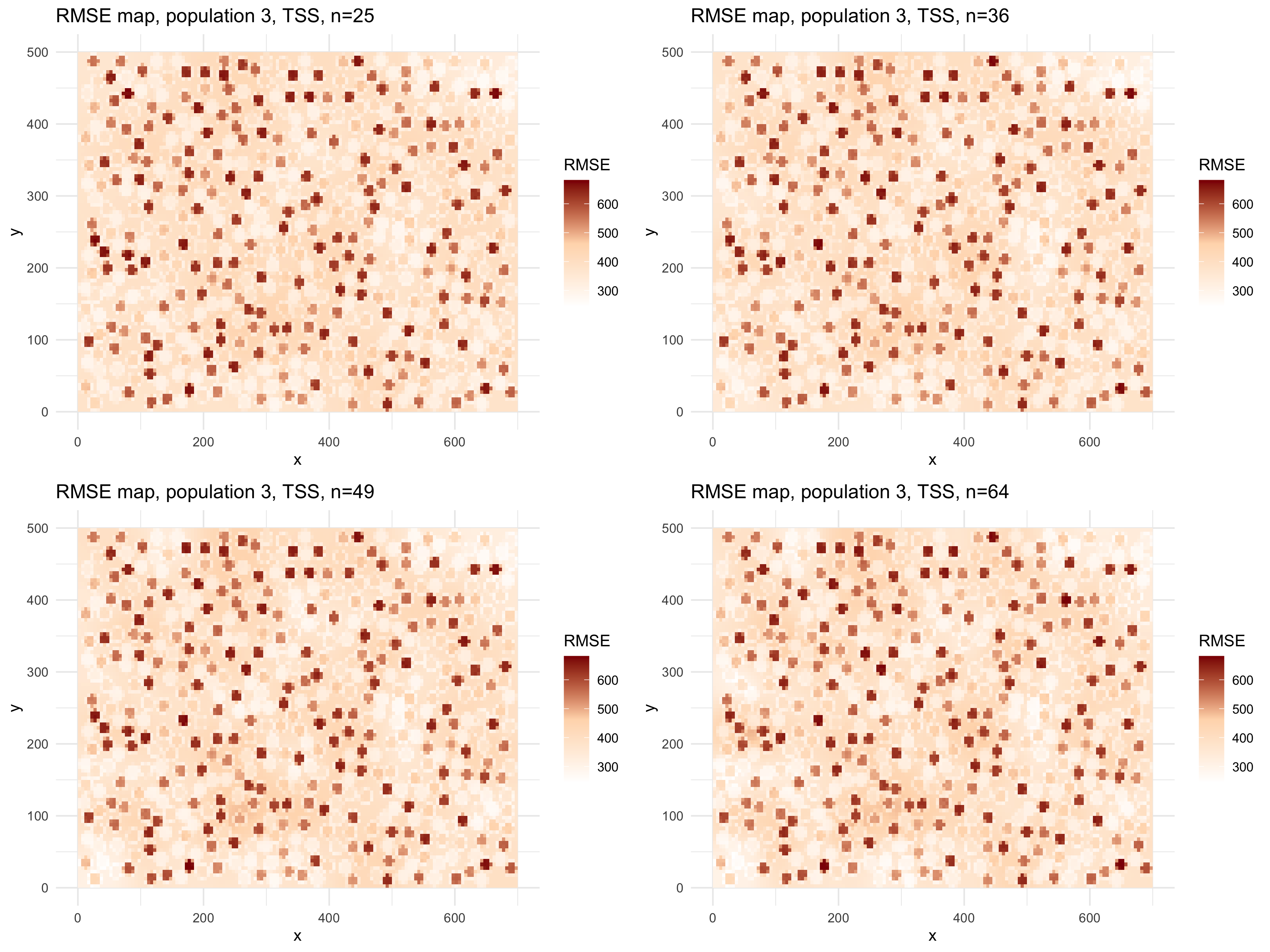** |

| **Figure SM21**: Population 3 bias map, URS, IDW interpolator |
| --- |
| 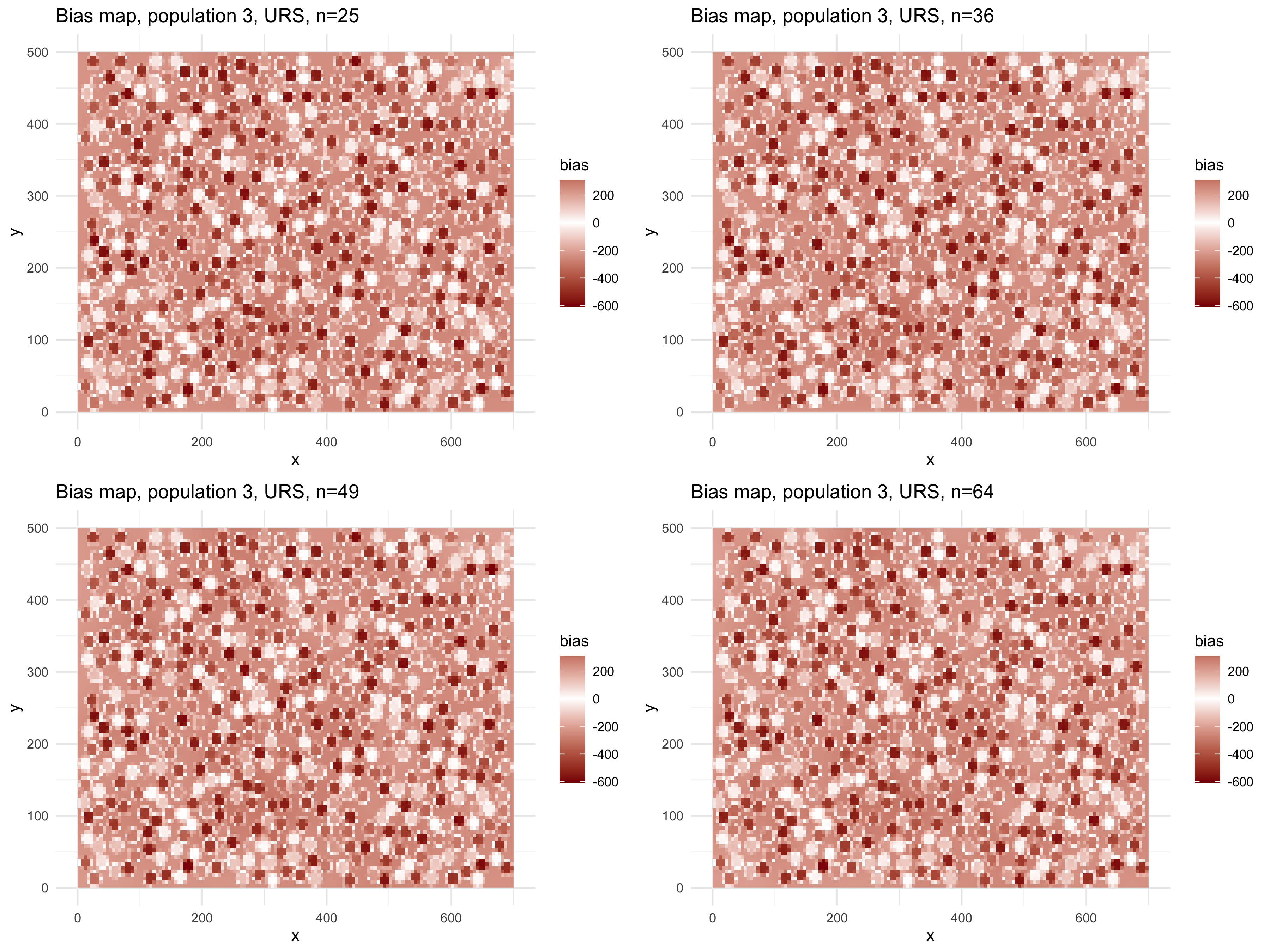 |
| **Figure SM22**: Population 3 RMSE map, URS, IDW interpolator |
| 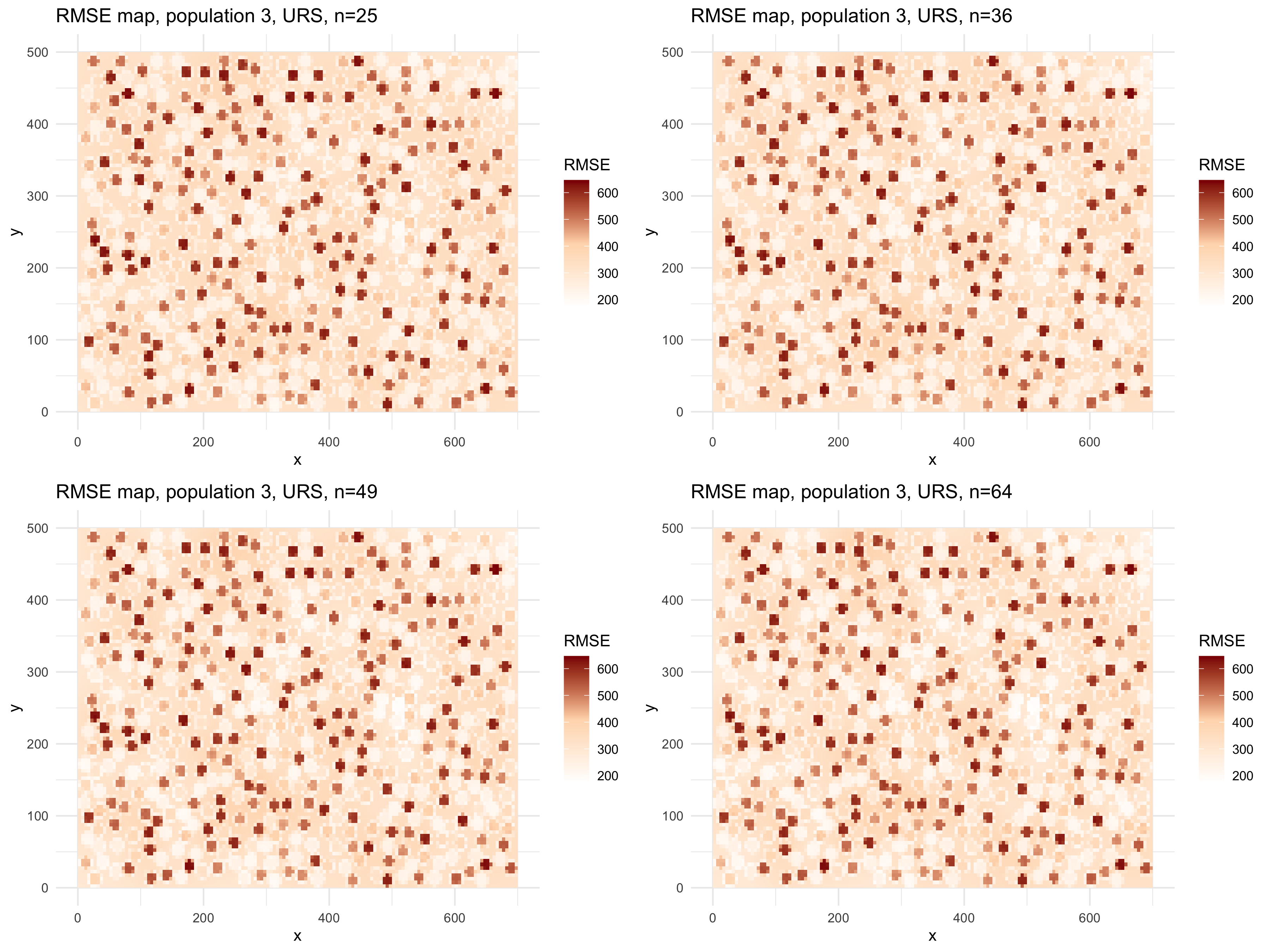 |

| **Figure SM23**: Population 3 bias map, TSS, IDW interpolator |
| --- |
| **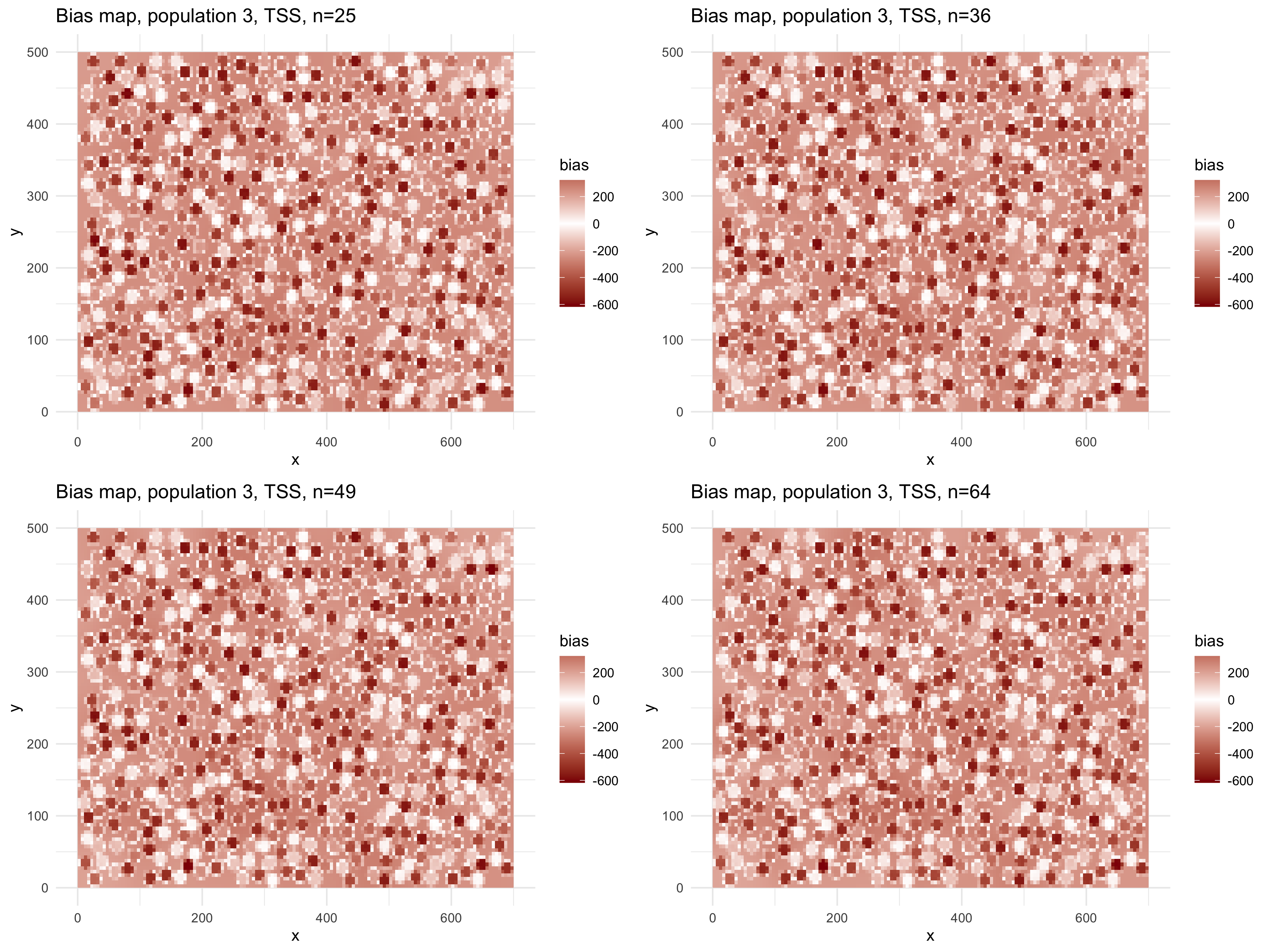** |
| **Figure SM24**: Population 3 RMSE map, TSS, IDW interpolator |
| **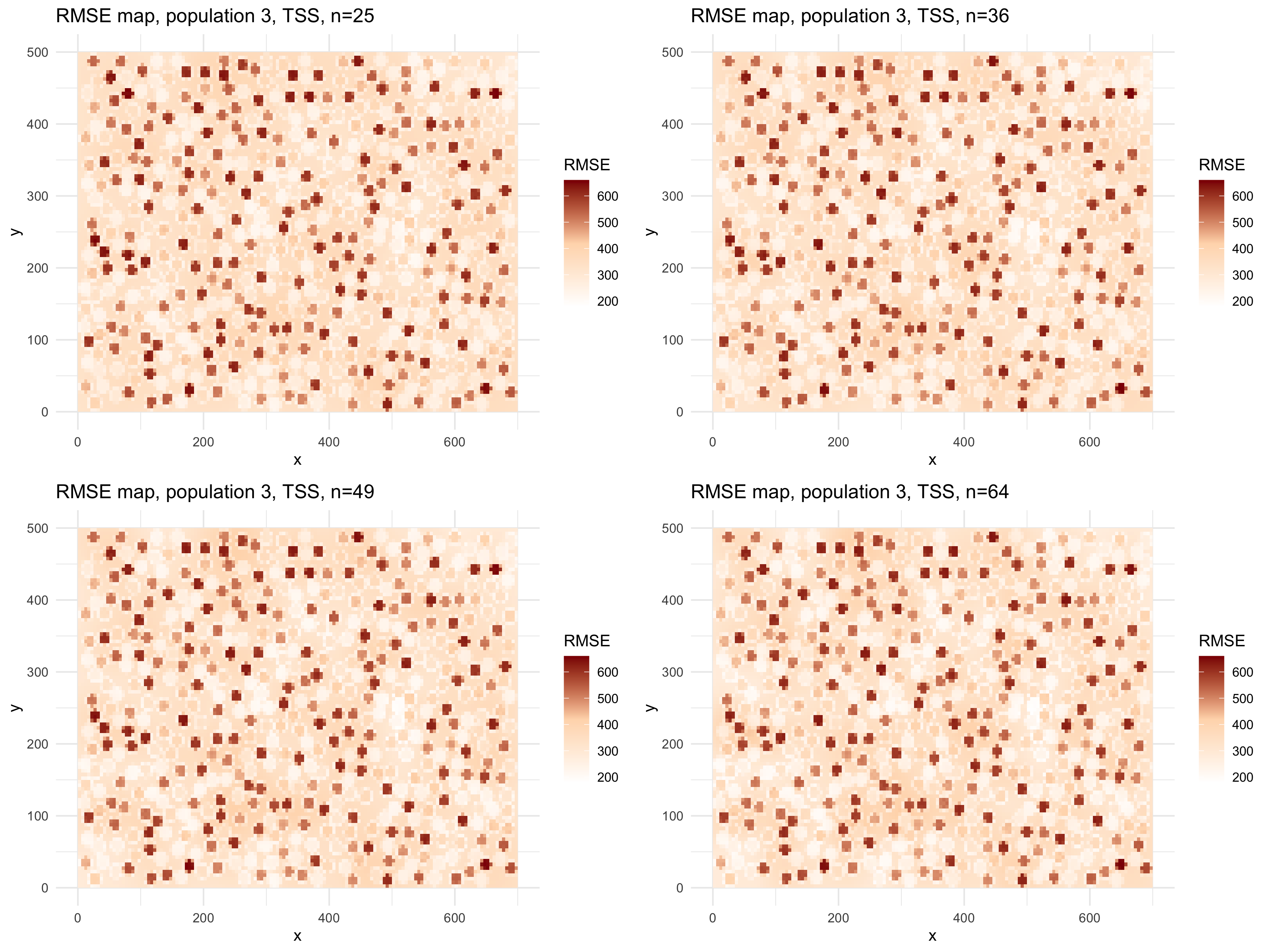** |

| **Figure SM25**: Population 4 bias map, URS, NN interpolator |
| --- |
| **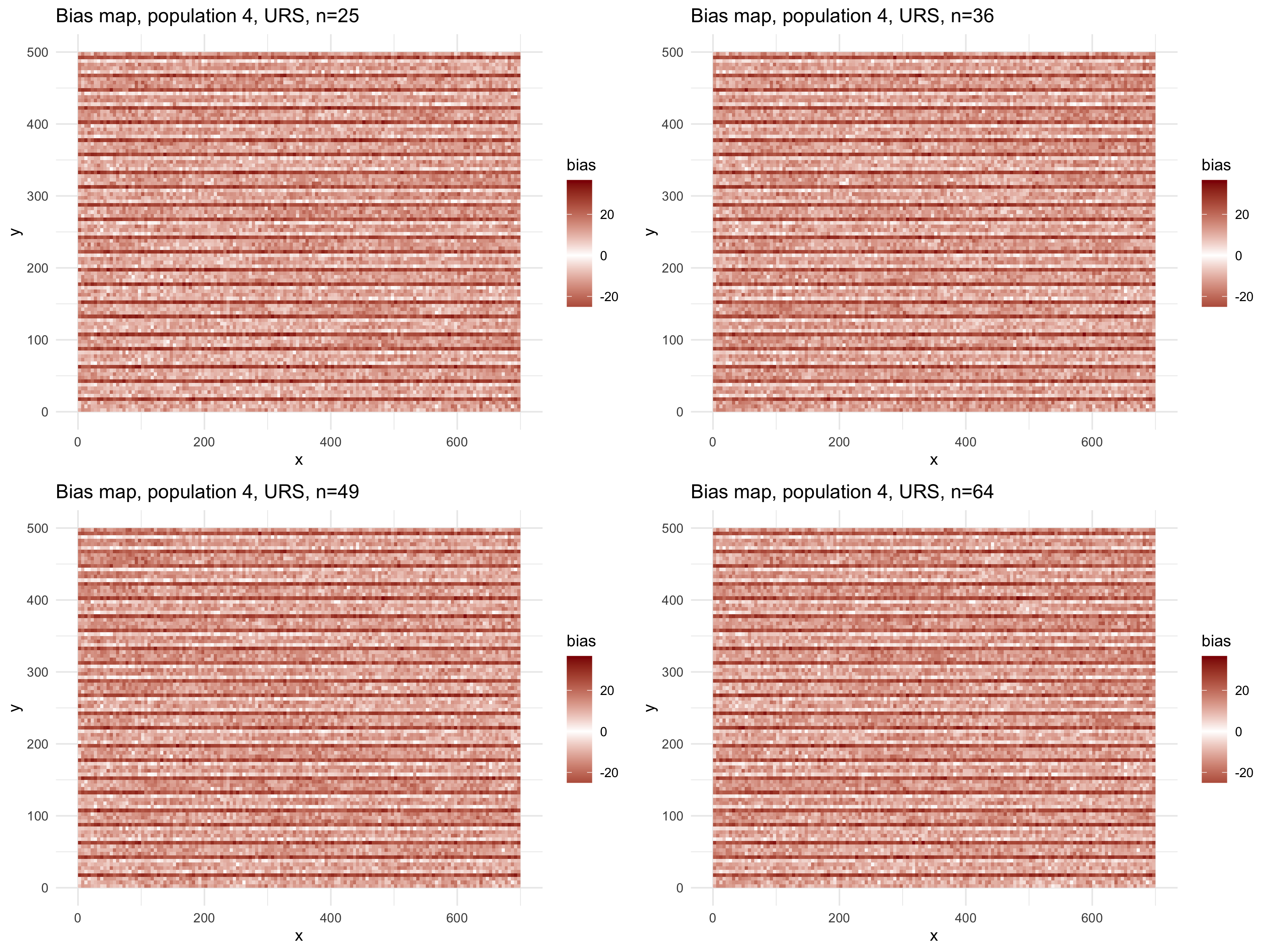** |
| **Figure SM26**: Population 4 RMSE map, URS, NN interpolator |
| **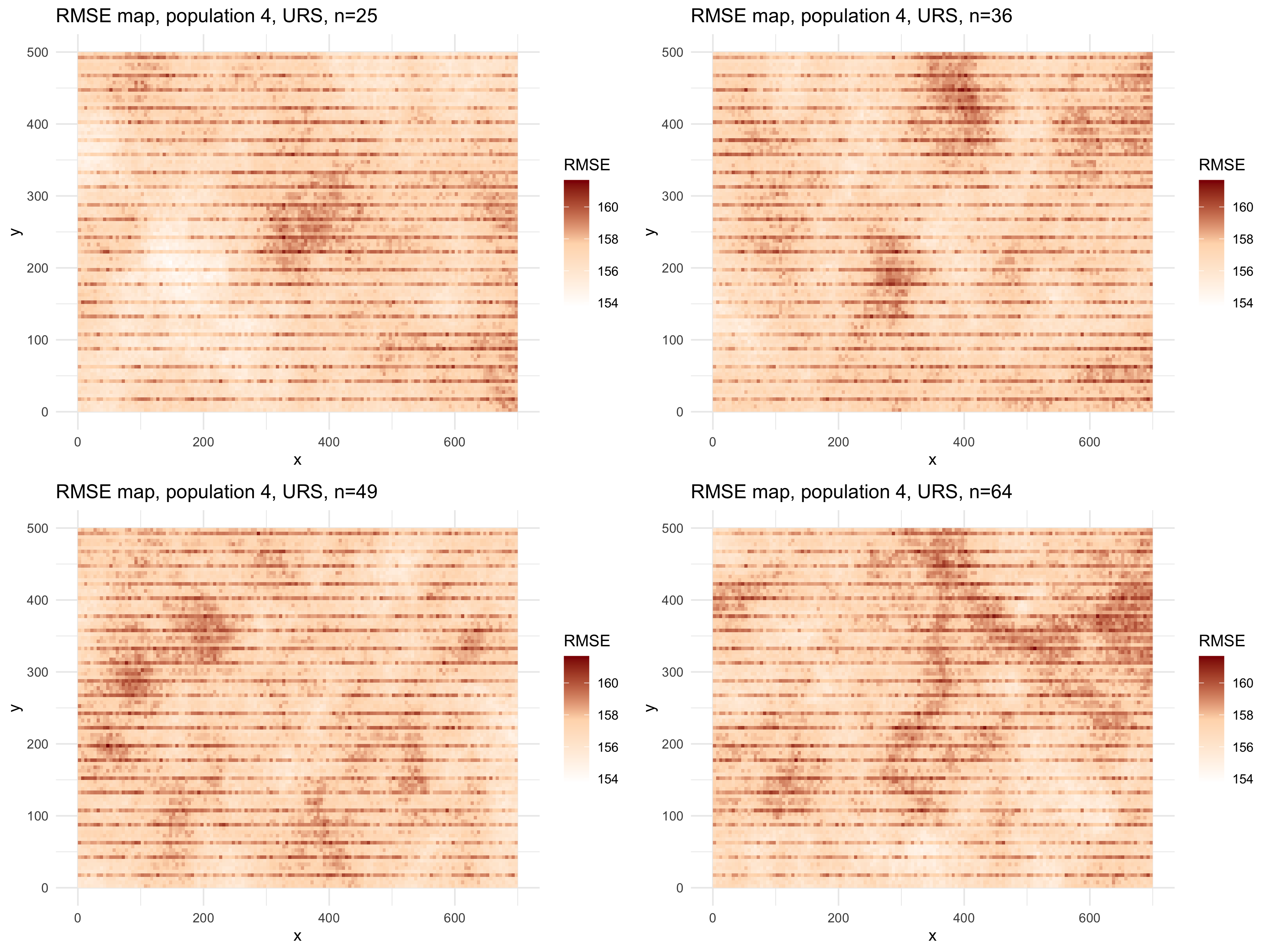** |

| **Figure SM27**: Population 4 bias map, TSS, NN interpolator |
| --- |
| 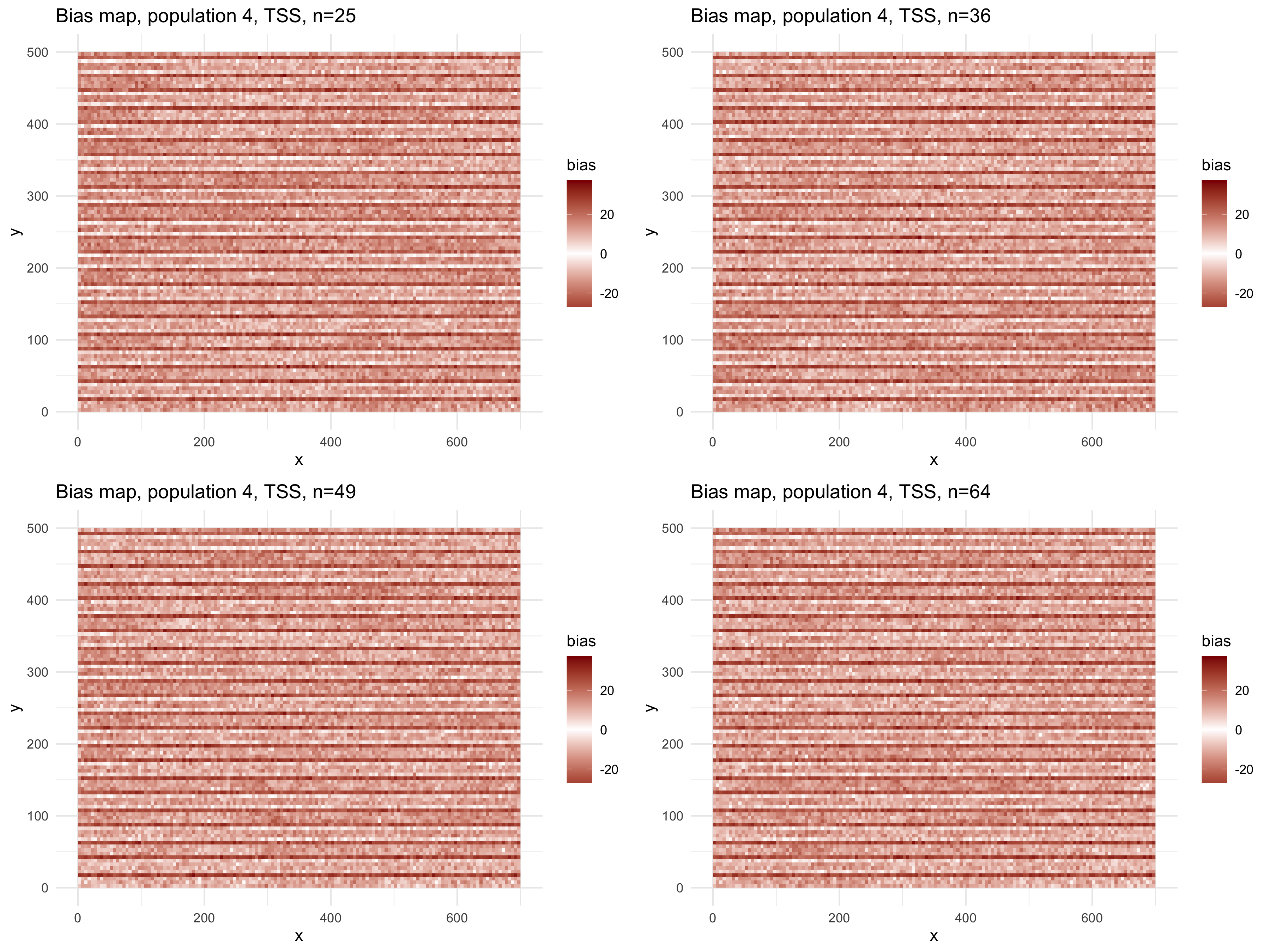 |
| **Figure SM28**: Population 4 RMSE map, TSS, NN interpolator |
| 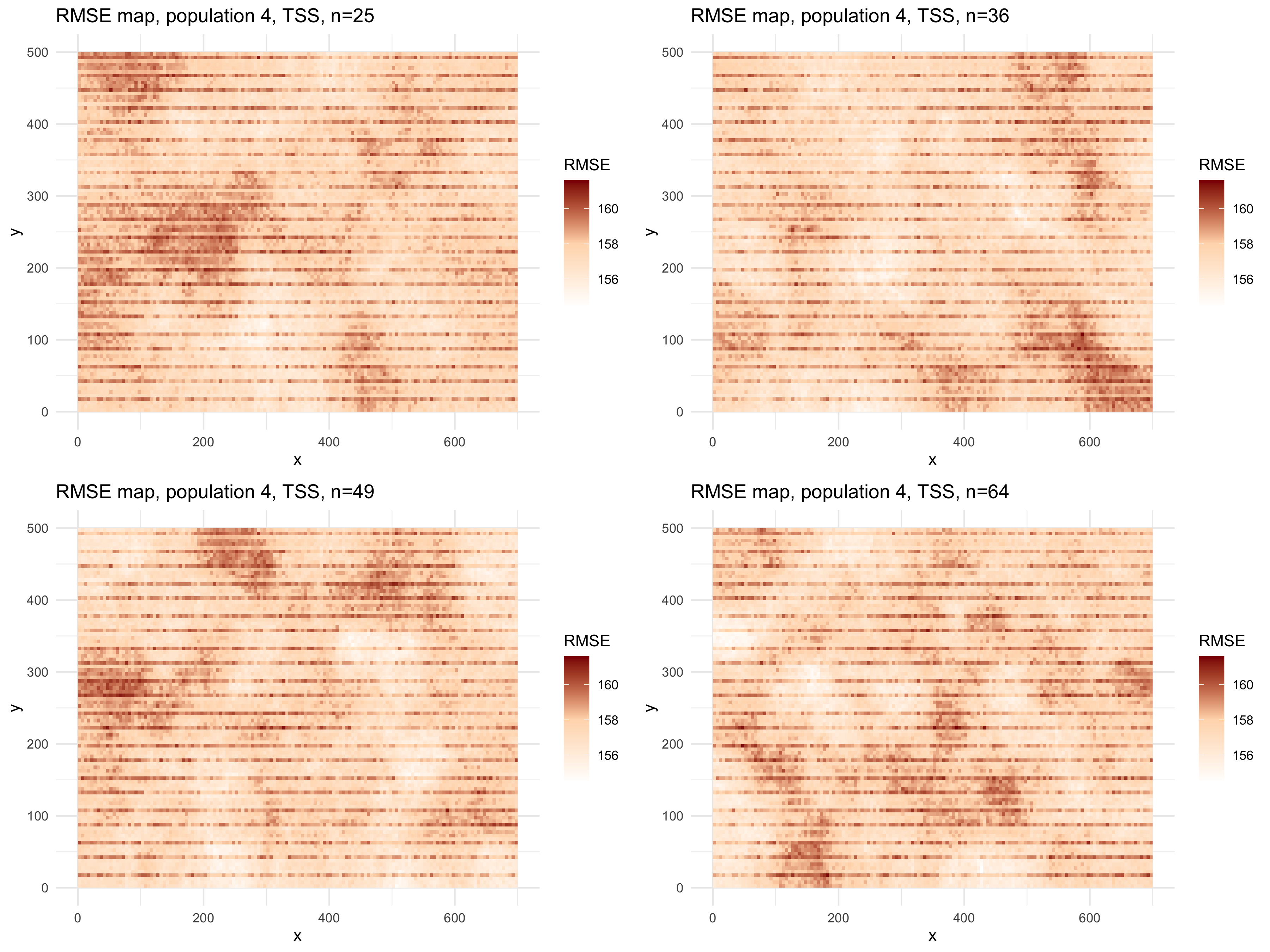 |

| **Figure SM29**: Population 4 bias map, URS, IDW interpolator |
| --- |
| 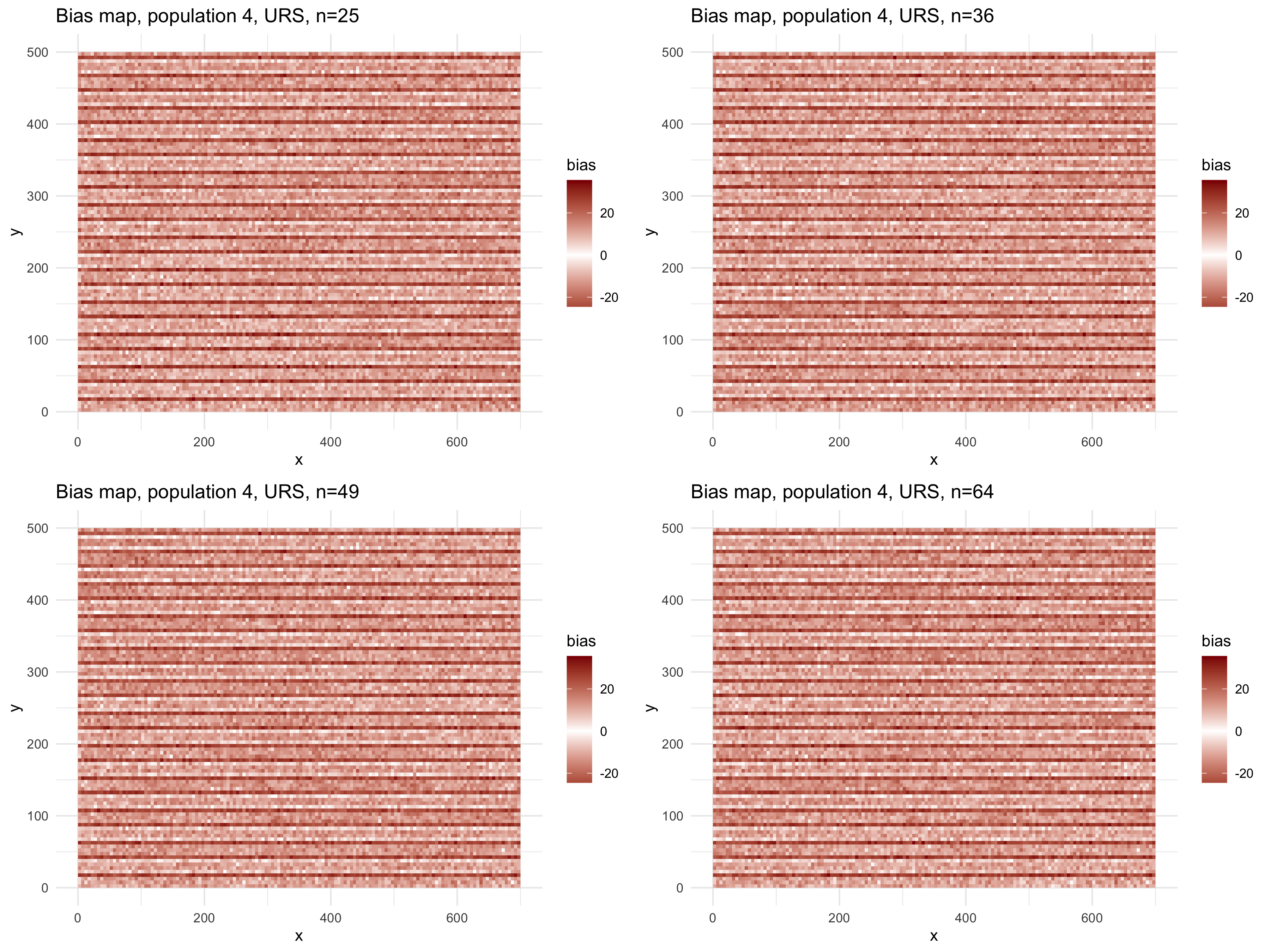 |
| **Figure SM30**: Population 4 RMSE map, URS, IDW interpolator |
| 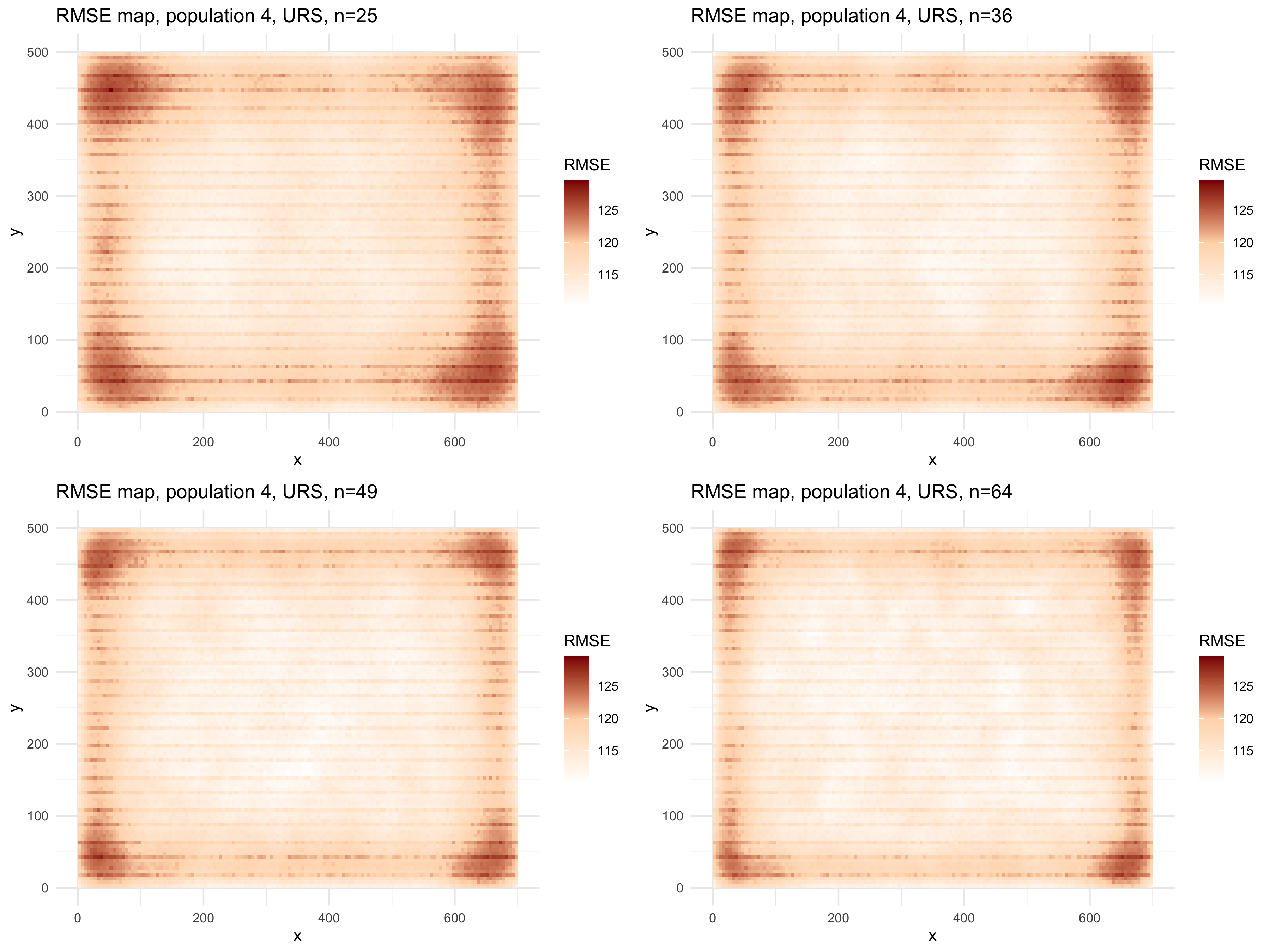 |

| **Figure SM31**: Population 4 bias map, TSS, IDW interpolator |
| --- |
| 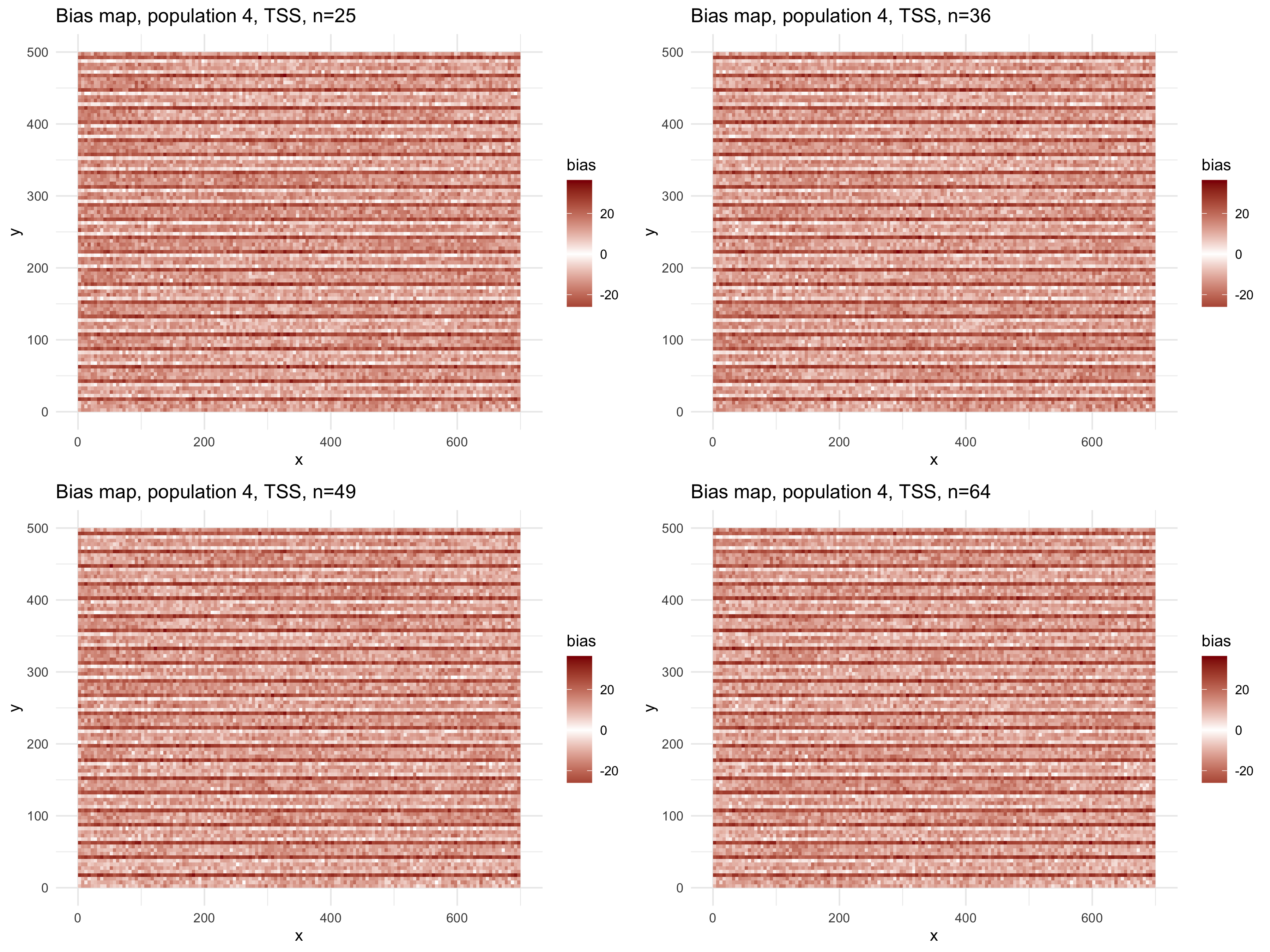 |
| **Figure SM32**: Population 3 RMSE map, TSS, IDW interpolator |
| 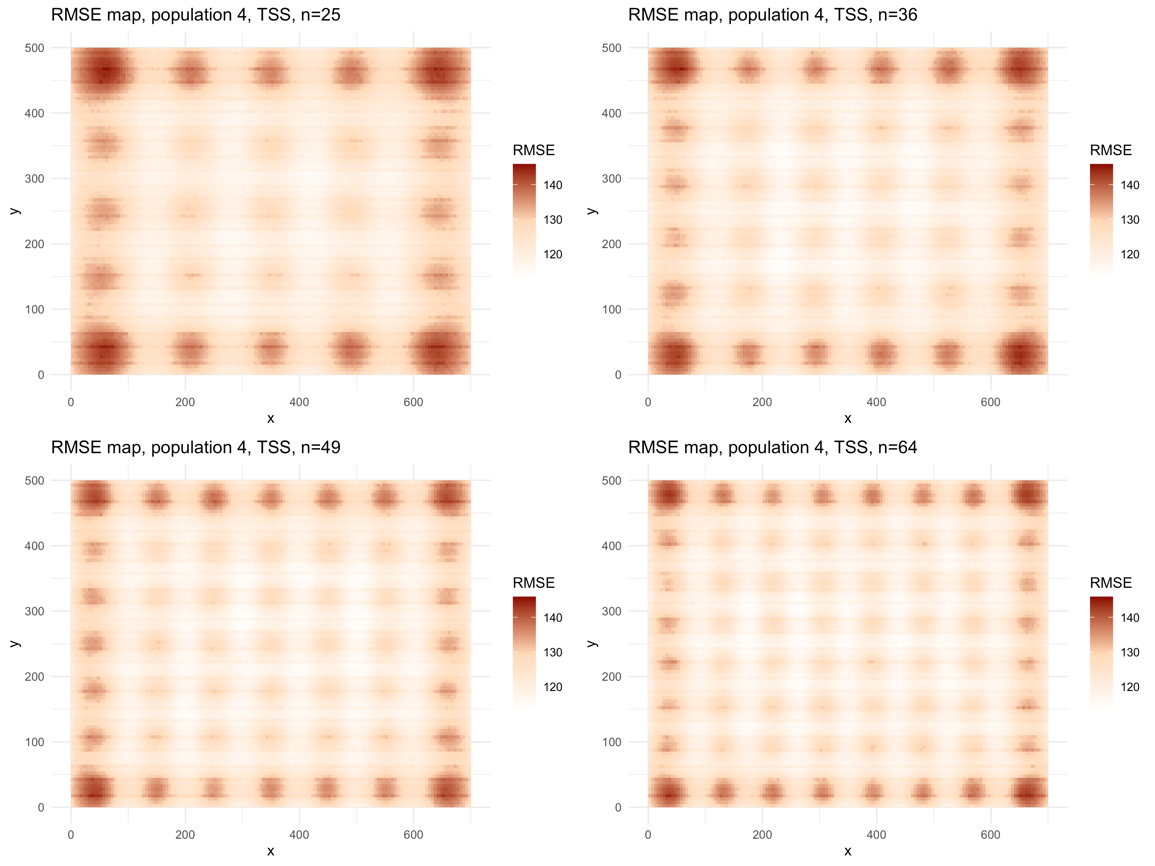 |
